# *Brucella* Induces an Unfolded Protein Response via TcpB That Supports Intracellular Replication in Macrophages

**DOI:** 10.1371/journal.ppat.1003785

**Published:** 2013-12-05

**Authors:** Judith A. Smith, Mike Khan, Diogo D. Magnani, Jerome S. Harms, Marina Durward, Girish K. Radhakrishnan, Yi-Ping Liu, Gary A. Splitter

**Affiliations:** 1 Department of Pediatrics, University of Wisconsin-Madison School of Medicine and Public Health, Madison, Wisconsin, United States of America; 2 Cellular and Molecular Pathology Training Program, University of Wisconsin-Madison, Madison, Wisconsin, United States of America; 3 Department of Pathobiological Sciences, University of Wisconsin-Madison School of Veterinary Medicine, Madison, Wisconsin, United States of America; 4 National Institute of Animal Biotechnology, Hyderabad, India; Yale University School of Medicine, United States of America

## Abstract

*Brucella melitensis* is a facultative intracellular bacterium that causes brucellosis, the most prevalent zoonosis worldwide. The *Brucella* intracellular replicative niche in macrophages and dendritic cells thwarts immune surveillance and complicates both therapy and vaccine development. Currently, host-pathogen interactions supporting *Brucella* replication are poorly understood. *Brucella* fuses with the endoplasmic reticulum (ER) to replicate, resulting in dramatic restructuring of the ER. This ER disruption raises the possibility that *Brucella* provokes an ER stress response called the Unfolded Protein Response (UPR). In this study, *B. melitensis* infection up regulated expression of the UPR target genes BiP, CHOP, and ERdj4, and induced XBP1 mRNA splicing in murine macrophages. These data implicate activation of all 3 major signaling pathways of the UPR. Consistent with previous reports, XBP1 mRNA splicing was largely MyD88-dependent. However, up regulation of CHOP, and ERdj4 was completely MyD88 independent. Heat killed *Brucella* stimulated significantly less BiP, CHOP, and ERdj4 expression, but induced XBP1 splicing. Although a *Brucella* VirB mutant showed relatively intact UPR induction, a TcpB mutant had significantly compromised BiP, CHOP and ERdj4 expression. Purified TcpB, a protein recently identified to modulate microtubules in a manner similar to paclitaxel, also induced UPR target gene expression and resulted in dramatic restructuring of the ER. In contrast, infection with the TcpB mutant resulted in much less ER structural disruption. Finally, tauroursodeoxycholic acid, a pharmacologic chaperone that ameliorates the UPR, significantly impaired *Brucella* replication in macrophages. Together, these results suggest *Brucella* induces a UPR, via TcpB and potentially other factors, that enables its intracellular replication. Thus, the UPR may provide a novel therapeutic target for the treatment of brucellosis. These results also have implications for other intracellular bacteria that rely on host physiologic stress responses for replication.

## Author Summary

*Brucella melitensis* is an intracellular bacterium that invades and replicates within macrophages and dendritic cells. With over 500,000 new infections per year, brucellosis is the most prevalent zoonosis worldwide and incurs significant human morbidity and economic loss. The intracellular location of *Brucella* renders the organism resistant to antibiotics. A safe and effective human vaccine does not exist. Thus, better understanding of the host-pathogen interactions supporting establishment of the intracellular replicative niche is critical. In this study, we found that infection of macrophages with *Brucella* induces a host stress response called the Unfolded Protein Response (UPR), a conserved stress response originating in the endoplasmic reticulum (ER). Full induction of the UPR requires live bacteria and expression of a microtubule modulating protein, TcpB. Inhibition of the UPR with the drug tauroursodeoxycholic acid significantly diminished *Brucella* replication. Together these results suggest *Brucella* induces the UPR to enable its own replication within host macrophages. Thus the UPR may represent a novel therapeutic target for the treatment of brucellosis.

## Introduction

Brucellosis is a chronic debilitating disease with protean manifestations and insidious onset most frequently caused by the facultative intracellular bacteria *Brucella melitensis* [1]. With over 500,000 new infections per year, brucellosis is the most prevalent zoonosis worldwide [2]. Brucellosis is most often acquired by consumption of contaminated dairy products. Following ingestion, *Brucella* infects macrophages and dendritic cells that constitute the replicative reservoir [3]. The intracellular replicative niche thwarts immune surveillance, complicates vaccine development, and renders the organism refractory to antibiotics [1]. A greater understanding of host-pathogen interactions is critical for elucidating disease pathogenesis and thus improving therapeutic strategies.

*Brucella* establishes its stealthy intracellular lifestyle through virulence factors. *Brucella* expresses a weakly endotoxic smooth LPS that directs bacterial uptake via class A scavenger receptor in lipid rafts [4]–[6]. Inside macrophages, ∼90% of bacteria are killed within the first 4 h. However, some *Brucella*-containing vesicles (BCV) avoid end-stage lysosomes and ultimately fuse with the endoplasmic reticulum (ER) [7]. Fusion appears to involve an early ER to Golgi vesicular compartment, as GAPDH and the small GTPases Rab2 and Sar1 are essential for replication [8], [9]. Replicative BCV contain ER markers including calnexin, calreticulin and sec61β [7]. Correct trafficking and ultimately replication depend upon *de novo* bacterial protein expression following cellular infection. In particular, BCV acidification activates the type IV secretion system encoded by the VirB operon [10]. VirB mutant BCV fail to fuse with the ER and VirB mutants are greatly attenuated *in vivo* [7]. Within 48 h of infection, *Brucella* induces a marked reorganization of the ER with ER membrane accretion around replicating bacteria [7]. The mechanism by which *Brucella* disrupts ER structure is currently unknown. Somehow, the host cell adapts to this perturbation, as *Brucella* infection inhibits apoptosis. Although the bacterial factors leading to successful infection are beginning to be clarified, the host pathways supporting replication remain poorly understood.

The requirement for ER fusion and dramatic restructuring of the ER suggest *Brucella* most likely disrupts ER homeostasis. To cope with physiologic and stressful perturbations of ER function, cells mobilize a conserved stress response called the Unfolded Protein Response (UPR) [11]. The UPR is initiated when unfolded proteins within the ER excessively bind the chaperone BiP/glucose regulated protein (Grp)78, titrating it away from three primary ER membrane resident stress sensors, inositol requiring kinase 1 (IRE1), activating transcription factor (ATF6), and PKR-like endoplasmic reticulum kinase (PERK). IRE1 is both a kinase that phosphorylates targets such as Jun kinase (JNK), and an endonuclease that cleaves 26 nucleotides from the X-box binding protein 1 (XBP1) mRNA, thus removing a premature stop codon [12]. Spliced XBP1 mRNA encodes the full-length transcription factor. Upon release of BiP, ATF6 traffics from ER to Golgi, where site-specific proteases cleave it to an active transcription factor. PERK phosphorylates eukaryotic initiation factor 2α, resulting in global translational attenuation apart from select open reading frames (e.g. ATF4 mRNA). The three primary stress sensor-dependent biochemical pathways regulate the following: 1) UPR target gene transcription, including chaperones and co-chaperones (e.g. BiP and ERdj4) that increase folding capacity, 2) molecules involved in ER associated degradation and 3) pro-apoptotic factors such as C/EBP homologous protein (CHOP). The UPR exerts a profound effect on multiple cellular processes including autophagy, apoptosis, ER and Golgi biogenesis, and lipid and protein synthesis. If ER stress remains unresolved despite these adaptive measures, the UPR initiates apoptosis [11].

One study suggests the UPR may play a role in *Brucella* replication. *Brucella* replicate less efficiently in IRE1 knockdown insect cells and IRE1 deficient murine embryonic fibroblasts [13]. The IRE1 axis of the UPR regulates autophagy, which appears to support replication in non-phagocytic cells [14], [15]. The role of autophagy in supporting *Brucella* survival and replication efficiency in macrophages remains somewhat controversial, though compelling work implicates early autophagy pathway proteins in completion of the *Brucella* intracellular life cycle [16], [17]. Serum starvation enhances bacterial replication in HeLa cells, which may reflect a component of ER stress [18]. However, the relevance of the UPR to *Brucella* replication in physiologic host cells (e.g. macrophages) remains unknown.

Although viral manipulation of the UPR has been extensively studied, very little is known about the effect of bacterial infection on the host UPR. Evidence for UPR activation has been detected in histologic sections from patients infected with *M. tuberculosis* [19]. However, the relationship between infection and host response was not clear. In one report, intracellular bacteria *Francisella*, *Listeria*, and *Mycobacteria* induced XBP1 mRNA splicing via toll like receptor (TLR) signaling [20]. Deficiency of the TLR-adaptor protein myeloid differentiation primary response gene 88 (MyD88) ablated TLR2 and decreased TLR4-dependent XBP1 splicing. TLR-dependent XBP1 splicing was not accompanied by downstream UPR target gene induction, although there was evidence supporting a role for XBP1 in synergistic cytokine induction. XBP1 was essential for optimal cytokine production and immune control of *Francisella in vivo*. The exact mechanism underlying this selective XBP1 pathway activation is unknown. Extracellular *Listeria monocytogenes* has also been shown to induce a more complete UPR, involving all three signaling axes, via production of listeriolysin [21]. However, the intracellular life cycle of this bacterium differs greatly from *Brucella*.

In this study, we evaluated induction of the host UPR by *Brucella* infection in macrophages. We detected activation of all three axes of the UPR, stemming from activation of IRE1, PERK and ATF6, as evident by increased UPR target gene expression and XBP1 mRNA splicing. Although XBP1 splicing appeared to be largely MyD88-dependent, UPR gene expression was independent of the TLR-signaling adaptor molecule. Optimal UPR target gene induction required both live bacteria and expression of the microtubule-modulating *Brucella* protein TcpB. Finally, tauroursodeoxycholic acid (TUDCA), a pharmacologic chaperone that inhibits the UPR, substantially decreased replication. Together these data suggest *Brucella* actively induces a UPR that enables its intracellular replication within the ER in macrophages.

## Results

### *Brucella* induces the UPR

**Figure 1:**
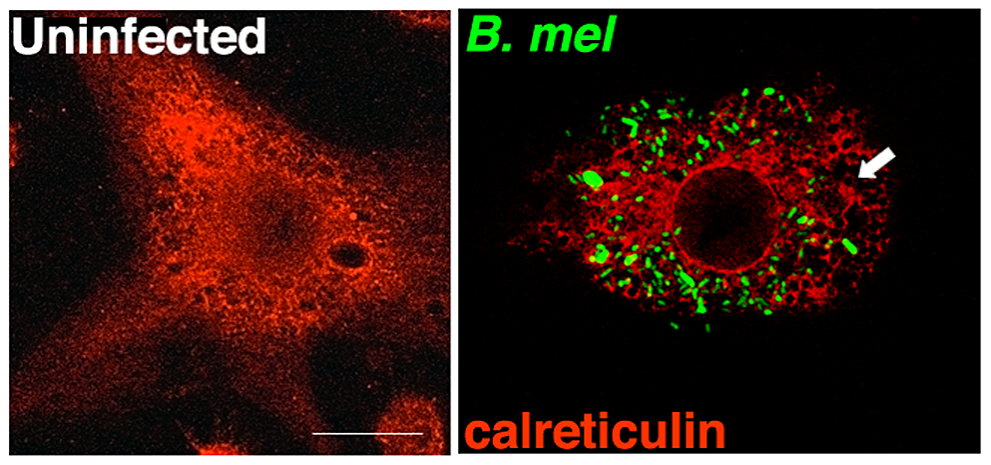
*Brucella* infection of macrophages induces ER structural reorganization. RAW 264.7 macrophages were uninfected or infected with YFP expressing *B. melitensis* (green) for 24 h. Arrow indicates condensation and fragmentation of the ER, visualized with anti-calreticulin antibody (red). Results are representative of 5 independent experiments.

**Figure 2:**
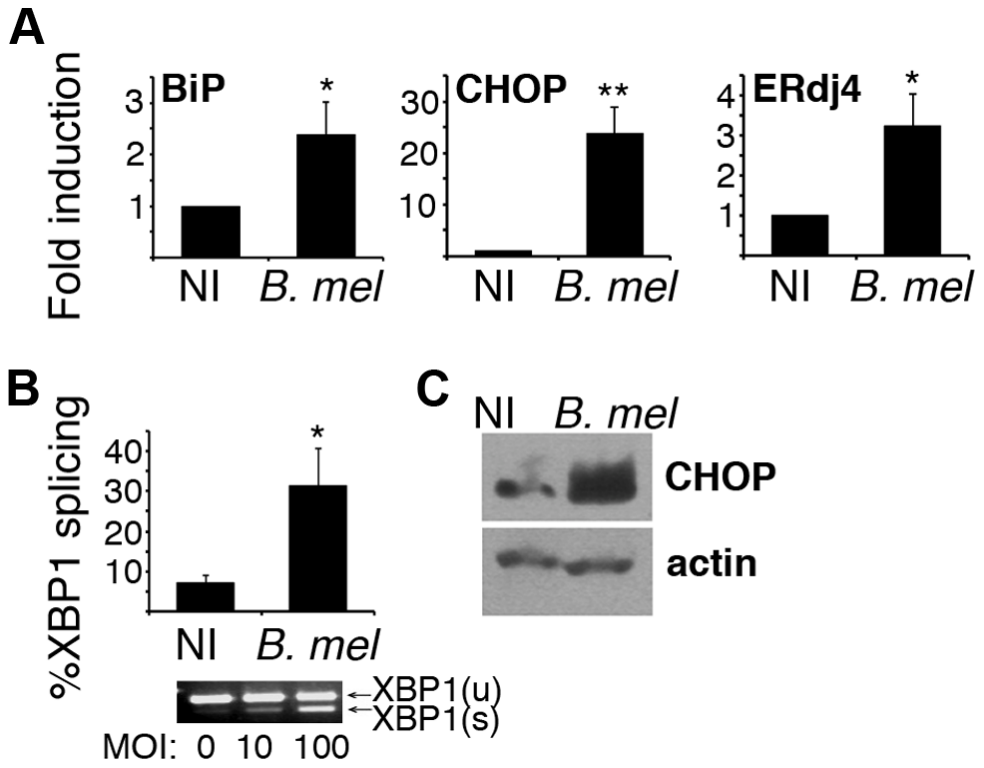
*Brucella* infection activates the UPR in macrophages *in vitro*. RAW 264.7 macrophages were uninfected (NI) or infected with 100 MOI *B. melitensis (B. mel)*. A) After 24 h, cells were resuspended in TRIzol for RNA processing. Relative expression of reverse transcribed cDNA was determined by quantitative PCR (qPCR) with normalization to 18S rRNA or hprt. Bars are combined mean fold inductions for 4–5 independent experiments (NI = 1) ± sem. *P<0.05, **p<0.003. B) Cells were infected with 100 MOI for 24 h and processed for RNA. XBP1 spliced and unspliced mRNA species were resolved by high-density agarose gel or detected by qPCR. %Splicing = spliced/total×100. Bars represent combined mean ± sem from 2–4 independent experiments. Representative gel is shown below. C) 16 h post infection, RAW cells were lysed and lysates resolved by SDS PAGE. CHOP or β-actin proteins were detected by immunoblot. Results are representative of 3 experiments.

A previous study documented dramatic reorganization of the ER within 48 h of *Brucella* infection [7]. We observed ER fragmentation and condensation even within 24 h of infection (Figure 1). The replicative requirement for ER-BCV fusion and the ER structural reorganization following infection raised the possibility that *Brucella* triggers the host cell UPR. The UPR directs an adaptive program through the induction of target gene transcription. Although the three primary biochemical signaling pathways have overlapping functions, several of the UPR gene targets appear to be relatively pathway specific; thus activation of PERK, IRE1, and ATF6 can be detected by downstream induction of mRNA for CHOP, ER localized DnaJ homologue 4 (ERdj4), and BiP, respectively [22]–[24]. XBP1 spliced and unspliced mRNA species can be resolved by high-percentage agarose gel electrophoresis, and this method is often used to detect IRE1 endonuclease activity [25]. To test for UPR activation, RAW264.7 macrophages were infected with *B. melitensis* for 24 h (Figure 2). Of the genes examined, CHOP showed the most robust induction and mRNA expression correlated with a marked increase in CHOP protein. The induction of BiP, CHOP and ERdj4 mRNA and evidence for XBP1 mRNA splicing supports the hypothesis that *Brucella* induces a UPR involving all three primary signaling axes in macrophages *in vitro*.

**Figure 3:**
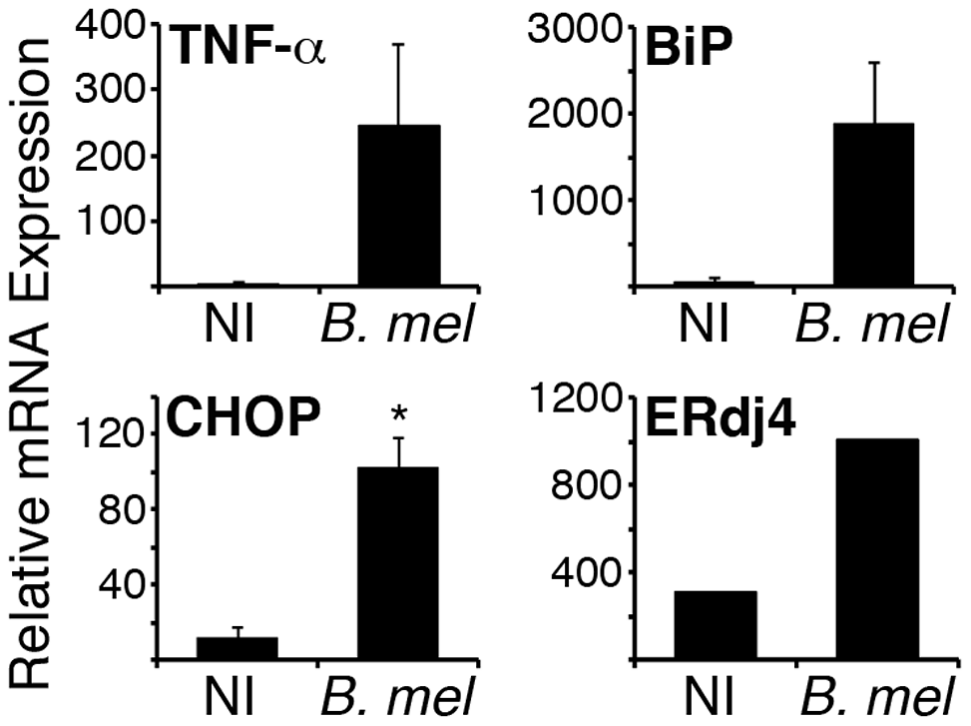
*Brucella* induces the UPR *in vivo*. BALB/c mice were injected ip with PBS (NI) or 10^7^ *B. melitensis* (*B. mel*). After 24 h, CD11b+ cells were isolated from pooled spleens and cells were resuspended in Trizol for RNA purification. TNF-α, BiP, CHOP, and ERdj4 gene expression was detected by qPCR with normalization to 18S rRNA. Error bars denote standard deviations between 2 pools (7 mice each). ERdj4 expression is from 1 pool each (NI or *B. mel*) of 4 mice. Results represent 2 independent experiments. *p = 0.01.

To determine if *Brucella* infection induces a detectable UPR *in vivo*, splenic CD11b+ cells (containing macrophages) were isolated 24 h following infection and UPR gene expression assessed by qPCR (Figure 3). TNF-α expression served as a positive control that is expected to increase early with infection. Induction of BiP, CHOP, and ERdj4 expression was evident following *in vivo* infection, consistent with activation of the UPR *in vivo*.

### *Brucella*-induced XBP1 mRNA splicing is MyD88 dependent

**Figure 4:**
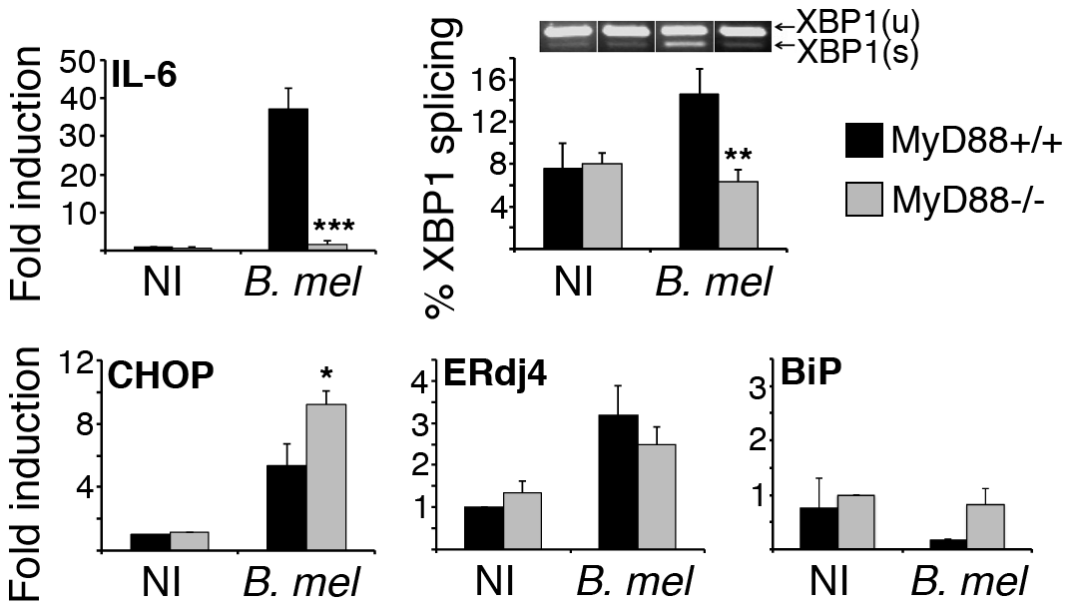
MyD88 deficiency impairs *Brucella* induced XBP1 splicing but not induction of other UPR genes. Bone marrow derived macrophages from MyD88−/− or wild type mice (MyD88+/+) were uninfected (NI) or infected with 100 MOI of *B. melitensis (B. mel)*. After 24 h, cells were processed for RNA isolation. Relative gene expression was determined by qPCR with normalization to 18S rRNA or hprt. Percent XBP1 splicing was quantified using Agilent and high-percent agarose gel. A sample agarose gel image shows corresponding unspliced and spliced (XBP1(u) and XBP1(s)) mRNA species. Data is combined from 3 (IL-6, BiP, XBP1 splicing) or 4 (CHOP, ERdj4) infected sets (one each) of wild type and MyD88−/− mice by normalizing fold induction to the non-infected control in each set (NI = 1). Error bars represent standard error of the mean. *P≤0.04 vs. infected MyD88+/+. **P≤0.02. ***P = 0.001.

A recent study described XBP1 splicing in response to TLR2 and TLR4 agonists (Pam3cysK4 and LPS) as well as various intracellular bacteria [20]. Interestingly, although XBP1 was required for optimal TLR-stimulated cytokine production, TLR ligation decreased BiP and CHOP induction by pharmacologic UPR inducers. Thus the TLR-MyD88-XBP1 pathway appears antagonistic towards the rest of the ER stress response. Another report also documents selective suppression of ER stress signaling by LPS [26]. *Brucella* stimulates both TLR2 and TLR4 and the TLR adaptor MyD88 appears to be essential for controlling infection *in vivo* [27]. To further elucidate the role of TLR-MyD88 signaling in UPR induction by *Brucella*, XBP1 splicing and UPR target gene expression was examined in primary bone marrow derived macrophages from MyD88 deficient mice (Figure 4). The phenotype of these mice was confirmed by diminished IL-6 expression following infection of macrophages *in vitro*. Although *Brucella* induced XBP1 mRNA splicing was impaired in MyD88−/− macrophages, induction of other UPR target genes, e.g. CHOP and ERdj4, was intact. In fact, induction of CHOP was slightly greater in the MyD88−/− macrophages (p = 0.034). This result is consistent with the described suppression of UPR target genes by TLR agonists. BiP expression was not upregulated at 24 h following infection in these experiments. XBP1 mRNA splicing in response to a pharmacologic UPR inducer, tunicamycin, was equivalent in the two mouse strains (82±1% in MyD88−/− vs 85±4% in wild type, data not shown). Together, these data suggest *Brucella* induces XBP1 splicing through TLR-MyD88 signaling; however induction of the other UPR target genes examined (CHOP and ERdj4) proceeds through a MyD88 independent pathway. These results further demonstrate *Brucella*-dependent UPR induction in primary macrophages, thus validating the RAW 264.7 macrophage cell line data.

### *Brucella* requirements for UPR target gene induction

**Figure 5:**
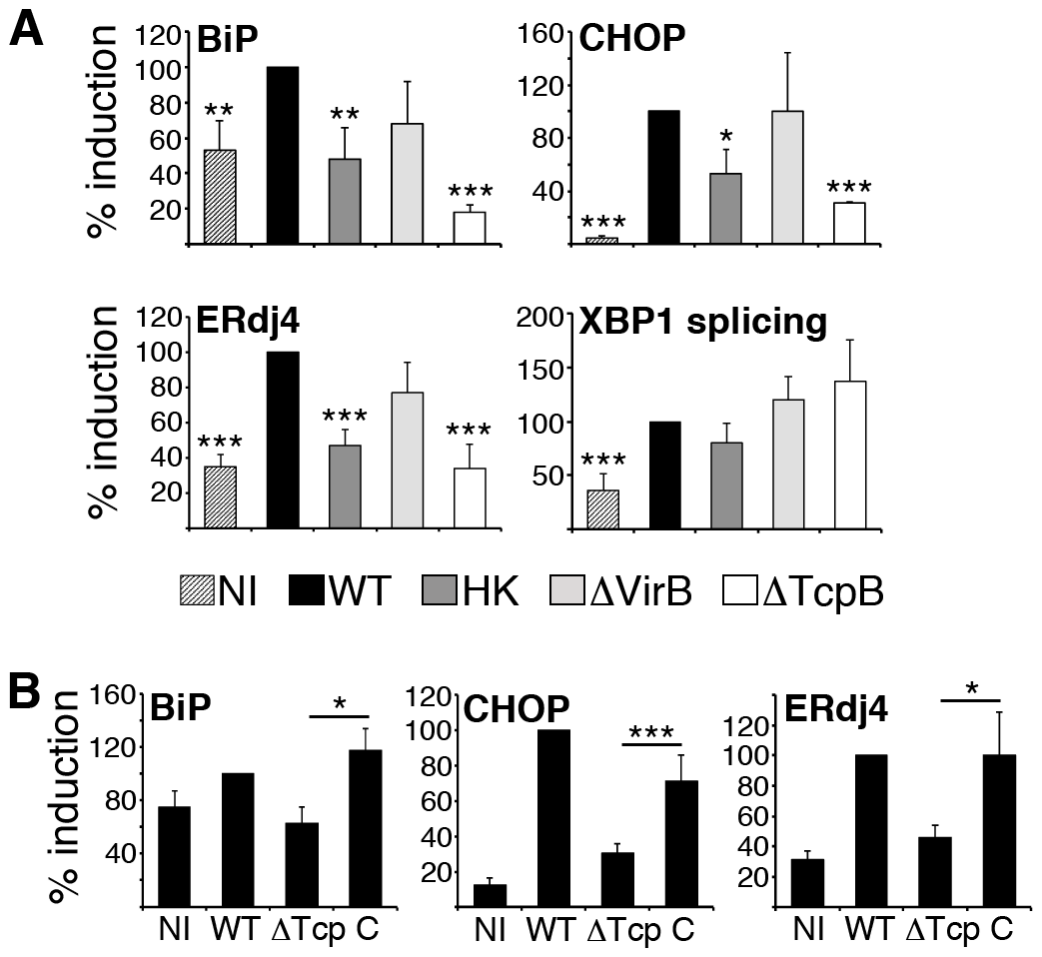
TcpB mutation reduces *Brucella*-induced UPR target gene expression. A) RAW 264.7 macrophages were uninfected (NI) or infected with 100 MOI *B. melitensis* (WT), heat killed *Brucella* (HK), a VirB deletion mutant (ΔVirB), or TcpB deletion mutant (ΔTcpB) for 24 h (legend at bottom of panel A). Cells were processed for RNA and relative UPR target gene expression (BiP, CHOP, and ERdj4) was determined by quantitative PCR (qPCR). XBP1 splicing was detected by qPCR. To combine independent experiments, WT induced UPR gene expression was set = 100%. Bars represent combined means of 3–4 (ΔTcpB) or 4 (HK, ΔVirB) experiments ± sem. *P<0.04, **p<0.02, ***p<0.009 vs. WT. B) RAW 264.7 or J774 cells were either uninfected (NI) or infected for 24 h with 100 MOI of 16M *B. melitensis* (WT), the TcpB deletion mutant (ΔTcp) or the complemented TcpB deletion mutant (C). RNA expression was normalized to 18S rRNA and WT 16M (set = 100%). Bars represent combined means of 14 (NI, WT) and 10 (ΔTcpB) experiments, and 5 complemented TcpB (C) mutant experiments. *P≤0.03, ***p = 0.008 comparing ΔTcpB mutant and complemented mutant gene expression.

One prediction of these data is that the bacterial surface of *Brucella* (containing LPS) will be sufficient to stimulate XBP1 splicing. However, the induction of other UPR-dependent events (e.g. increased CHOP expression) may involve other surface or intracellular components. To begin testing this premise, RAW macrophages were infected with heat killed *Brucella* (Figure 5A). Heat killed *Brucella* induced XBP1 splicing to a similar extent as living *Brucella*, as predicted, but induced significantly less UPR target gene expression. These data suggest other heat-labile or newly produced factors besides bacterial LPS are responsible for activating the host UPR.

The need for living bacteria for optimal UPR target gene induction may reflect the involvement of *de novo* bacterial protein/factor production following infection. *De novo* expression of virulence factors directs the distinctive trafficking and replicative events that result in chronic infection. In particular, products encoded in the VirB operon appear to be essential for fusion of BCV with ER membranes and subsequent replication [7]. To test the requirement for VirB, UPR induction was assessed in a VirB4 mutant (Figure 5A) [28]. This mutant displays attenuated virulence, with defects in *in vivo* persistence. Consistent with above results, XBP1 splicing was intact. Downstream UPR target gene induction following 24 h infection with the VirB deletion mutant (ΔVirB) was variable, and not statistically different compared to the wild type control. These data suggested another *Brucella* factor, besides those encoded by VirB, or utilizing the VirB-dependent type IV secretion system, must be involved in UPR induction.

### The role of TcpB in UPR gene induction and ER structural reorganization

The *B. abortus* protein Btp1 (*Brucella*-TIR-Protein 1) was originally characterized by its ability to inhibit dendritic cell maturation and to antagonize TLR2 signaling [29]. TcpB (Toll/Interleukin 1 like receptor domain containing protein), the correlating protein in *B. melitensis* also antagonizes TLR signaling and NF-κB activation [30]. We have recently shown that TcpB co-localizes with plasma membrane and microtubules and exerts a microtubule stabilizing effect similar to paclitaxel (Taxol) [31]. Besides co-localizing with cytoskeletal elements, exogenously expressed TcpB also co-localizes by immunofluorescence with the ER protein calreticulin (Figure S1). ER structure is microtubule dependent [32]. In the context of cancer research, microtubule-stabilizing agents such as paclitaxel have been shown to induce ER stress [33], [34]. Microtubules also regulate intracellular vesicular trafficking [35]. Brefeldin A, which blocks egress from the ER is commonly utilized to induce the UPR [36]. Thus we reasoned that TcpB might contribute to UPR induction through microtubule-related modification of ER structure. As shown in Figure 5A, infection with the TcpB deletion mutant (ΔTcpB) resulted in ≈60% decreased expression of BiP, CHOP and ERdj4 as compared to wild type *Brucella*. Note, some CHOP up-regulation by the TcpB mutant was still detectable (p≤0.005 vs. NI). Complementation of the TcpB mutant with exogenous TcpB recovered UPR gene expression (Figure 5B). These results were consistent with a role for TcpB protein in UPR induction.

**Figure 6:**
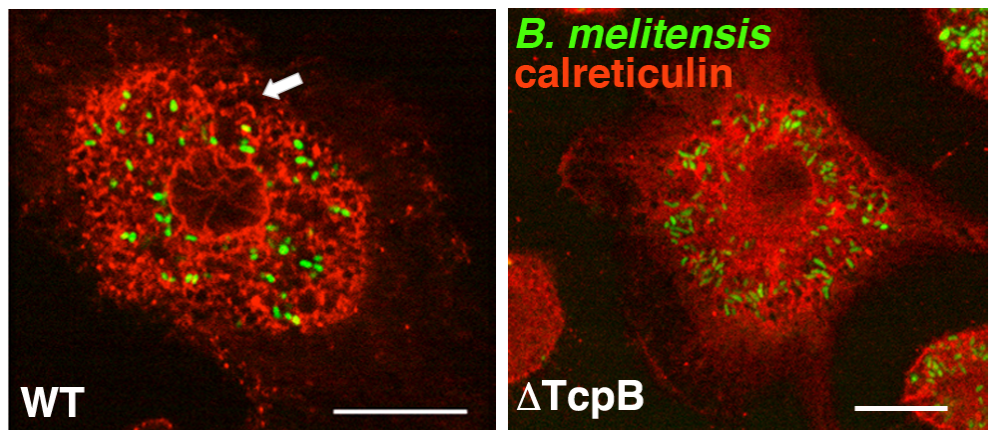
TcpB mutation results in less ER structural disruption following infection. RAW 264.7 cells were infected with an YFP-expressing TcpB deletion mutant (ΔTcpB) or wild type (WT) *B. melitensis* (green) for 24 h. The ER is visualized with anti-calreticulin (red). Arrow indicates ER condensation and fragmentation. Confocal microscopy results are representative of 5 independent experiments. Scale bar is 20 µM.

We hypothesized that UPR induction and ER restructuring are related events. In this case, the diminished UPR induction by the TcpB mutant should correlate with decreased effect on ER structure. Indeed, infection with the TcpB mutant did not induce the same degree of condensation and vacuolization as observed upon infection with wild type *Brucella* (Figure 6, Figure S2). The ER remains lacy, reticular and more evenly distributed compared to wild type infection. Trafficking of the *Brucella* within the cell however appears relatively intact, as the *Brucella* still migrate centrally to form a ring around the nucleus. Thus trafficking may not depend upon dramatic ER restructuring.

**Figure 7:**
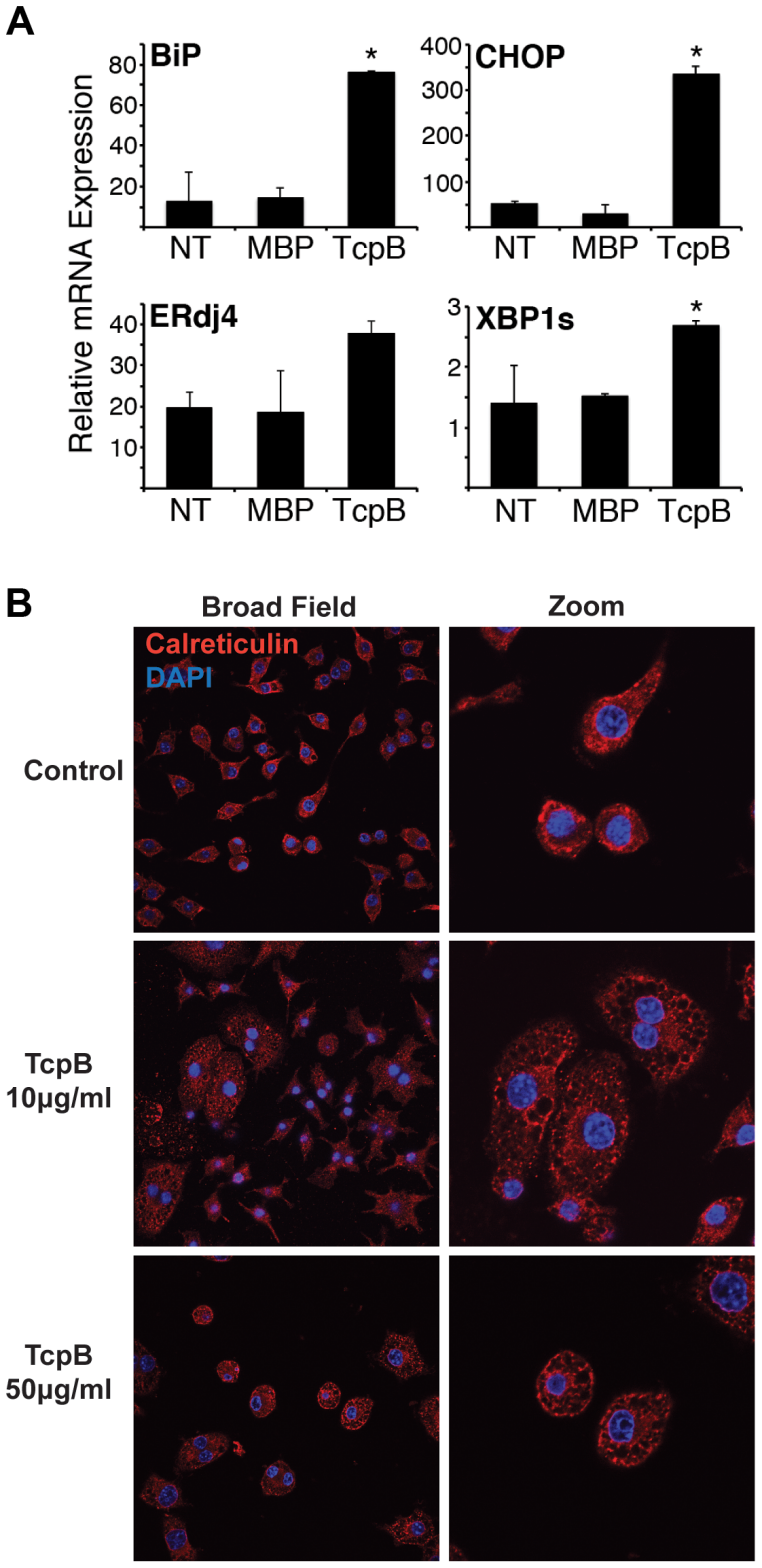
TcpB protein induces UPR and ER restructuring. A) RAW 264.7 cells were untreated (NT) or stimulated with 50 µg/mL MBP or MBP-TcpB (TcpB). After 24 h, cells were processed for RNA and relative expression of BiP, CHOP, ERdj4, or spliced XBP1 (XBP1s) determined by qPCR, with normalization to 18S rRNA. Bars depict combined means from 2 independent experiments, and are representative of 3 experiments, *p<0.004 vs. MBP. For ERdj4, p value is not significant vs. MBP, but P = 0.03 vs. non-treated cells. B) RAW 264.7 cells were untreated (Control), or stimulated with 10–50 µg/mL purified MPB-TcpB for 24 h. Cells were then fixed, stained for calreticulin, (red) and nuclei counterstained with DAPI (blue). Cells were imaged at 60× in close up (Zoom, right) or without additional digital zoom (Broad Field, left).

To directly test the role of TcpB in UPR induction and ER restructuring, RAW 264.7 macrophages were treated with purified TcpB protein using a concentration previously shown to affect microtubules and NF-κB signaling (Figure 7A) [30], [31]. TcpB protein was sufficient to upregulate BiP, CHOP, ERdj4 and spliced XBP1. The relative magnitude of effect appeared much greater for BiP and CHOP than for ERdj4 and spliced XBP1. Triggering of UPR events correlated well with effects of TcpB on ER structure as detected by immunofluorescence microscopy (Figure 7B). Compare the diffuse lacy reticular pattern extending throughout the cell in untreated or the MBP treated cells (Figure S3 and 7B) to the circumscribed circular area with large holes and more defined compact structures in TcpB treated cells. The majority of cells examined appeared similarly affected. Overall ER area appears enlarged, particularly at the lower dose of TcpB (Figure S4). ER condensation and fragmentation increases with dose of purified TcpB. Similar effects were observed by 12 h of treatment (not shown). This effect on ER structure was qualitatively similar to that induced by infection of macrophages with wild type *Brucella* (Figures 1 and 6). Together these results implicate TcpB in both ER fragmentation and UPR induction.

**Figure 8:**
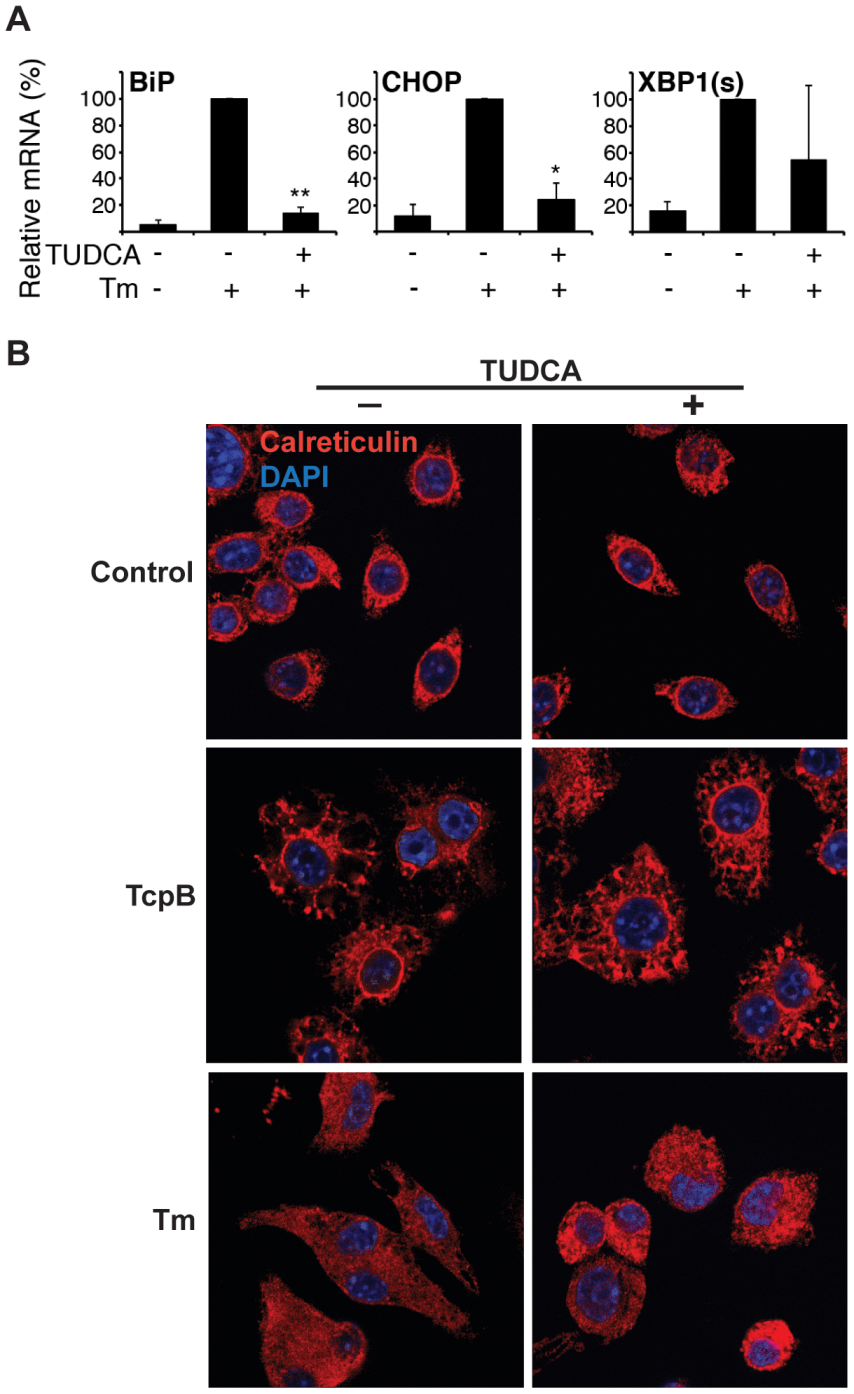
TcpB induced ER restructuring is not dependent on the UPR. A) RAW264.7 macrophages were pre-treated with 500 µg/mL TUDCA 30 min., followed by 6 h 10 µg/mL tunicamycin (Tm) as indicated, and then harvested for RNA. Relative UPR gene expression was assessed by qPCR. Results were combined from 2 independent experiments, *p = 0.01 and **p = 0.001 vs. Tm treatment only and NS = not significant vs. untreated cells. B) RAW 264.7 cells were pre-treated with 500 µg/mL TUDCA (TUDCA +) or not pre-treated (TUDCA −) for 60 min prior to stimulation with 50 µg/mL TcpB, 10 µg/mL tunicamycin (Tm), or media (Control). Cells were then fixed, stained for calreticulin (red), and counterstained with DAPI. Images are 100×.

It was unclear how the ER disruption related to the UPR. Were the ER structural changes a result of ER stress or is the UPR downstream of the ER disruption? To begin addressing this question, macrophages (or in some experiments D17 osteosarcoma cells) were treated with the ER stress inducer tunicamycin, a potent N-linked glycosylation inhibitor (Figure 8) [37], [38]. Although tunicamycin caused ER vacuolization, most likely related to proteins being retained in the ER, the disposition of ER calreticulin in the cell was different compared to TcpB treatment (or infection, see above): in the tunicamycin treated cells, the ER did not condense in a sphere but remained distributed into the macrophage processes. Thus TcpB induced disruption does not simply reiterate an ER stressor. If TcpB-induced ER restructuring were upstream of the UPR and not dependent on UPR, then blockade of the UPR should have no effect on ER disruption. To address this hypothesis, we inhibited the UPR with tauroursodeoxycholic acid (TUDCA), a chemical chaperone widely utilized *in vitro* and *in vivo* to modulate the UPR. The ability of TUDCA to impede BiP and CHOP induction by tunicamycin was confirmed (Figure 8). Inhibition of XBP1 splicing was more variable. TUDCA also inhibited tunicamycin-dependent cytokine induction (Figure S5) as expected. TUDCA treatment mitigated the effect of tunicamcyin (less vacuolization and size increase) but had no apparent effect on TcpB-related ER restructuring. These results suggest that ER restructuring is not UPR dependent. If ER structure and UPR are interdependent, ER disruption must occur upstream of UPR induction.

### UPR blockade inhibits *Brucella* replication

**Figure 9:**
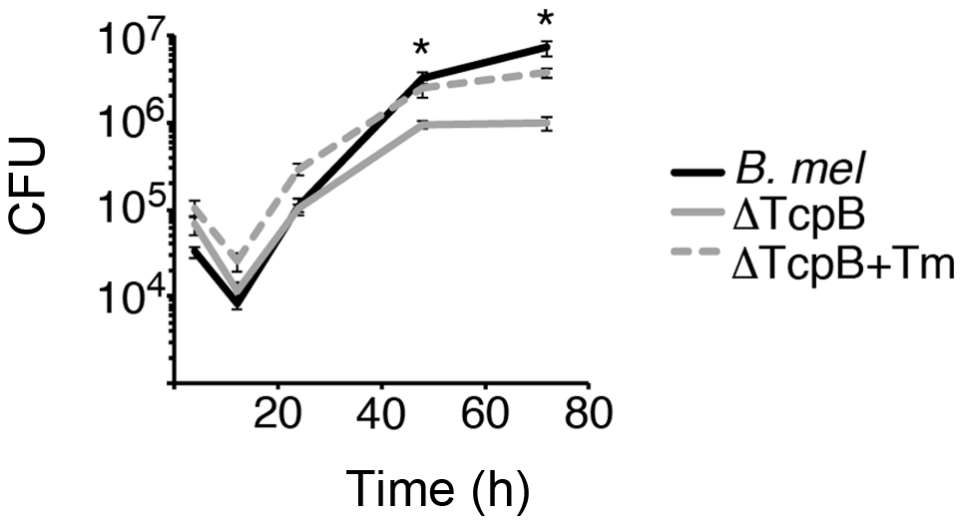
The TcpB mutant displays altered growth in macrophages. RAW264.7 macrophages were infected with wild type (*B. mel*, black) or TcpB mutant *Brucella* (ΔTcpB, gray). Select TcpB mutant cultures were also pre-treated with tunicamycin at 0.01 µg/mL (dashed lines). At different times following infection, cells were lysed and CFU determined by transfer to dilution plates. Error bars depict standard deviations of quadruplicate determinations. *P≤0.001. Results are representative of 4 independent experiments showing altered late growth of ΔTcpB.

The above data suggests *Brucella* induces the UPR at least in part via TcpB. However, it was not clear if the host mounts a UPR in response to infection, or if the UPR benefits the bacteria (or both). Viral infections manipulate the UPR in a variety of ways, including capitalizing on host protein production and folding machinery to enhance replication. One report utilizing insect cells and mouse embryonic fibroblasts suggests the IRE1 branch of the UPR supports *Brucella* replication, but the relevance to macrophages was unclear [13]. *Brucella* may not behave exactly the same in macrophages and non-phagocytic cells [15], [39]. TcpB mutant *Brucella* are defective at spreading systemically early during infection *in vivo* [27]. However, the other effects of TcpB, in particular inhibition of TLR signaling in the setting of an *in vivo* immune response, complicate the interpretation. To determine if TcpB plays a role in intracellular replication in macrophages *in vitro*, RAW 264.7 cells were infected with wild type *B. melitensis* or the TcpB mutant. Select cultures were also treated with very low dose tunicamycin to enhance the UPR (Figure 9). Initial uptake of the TcpB mutant was greater than wild type (p = 0.008), but the replication growth curve plateaus below the level observed in wild type. This slowed growth resulted in decreased CFU later during the culture period (p≤0.001 after 24 h). Tunicamycin treatment enhanced recoverable TcpB mutant CFU at all time points (p = 0.04 at 4 h and p≤0.006 thereafter). This effect of tunicamycin is consistent with previous reports documenting enhanced *Brucella* replication by serum starvation (nutrient deprivation) [18].

**Figure 10:**
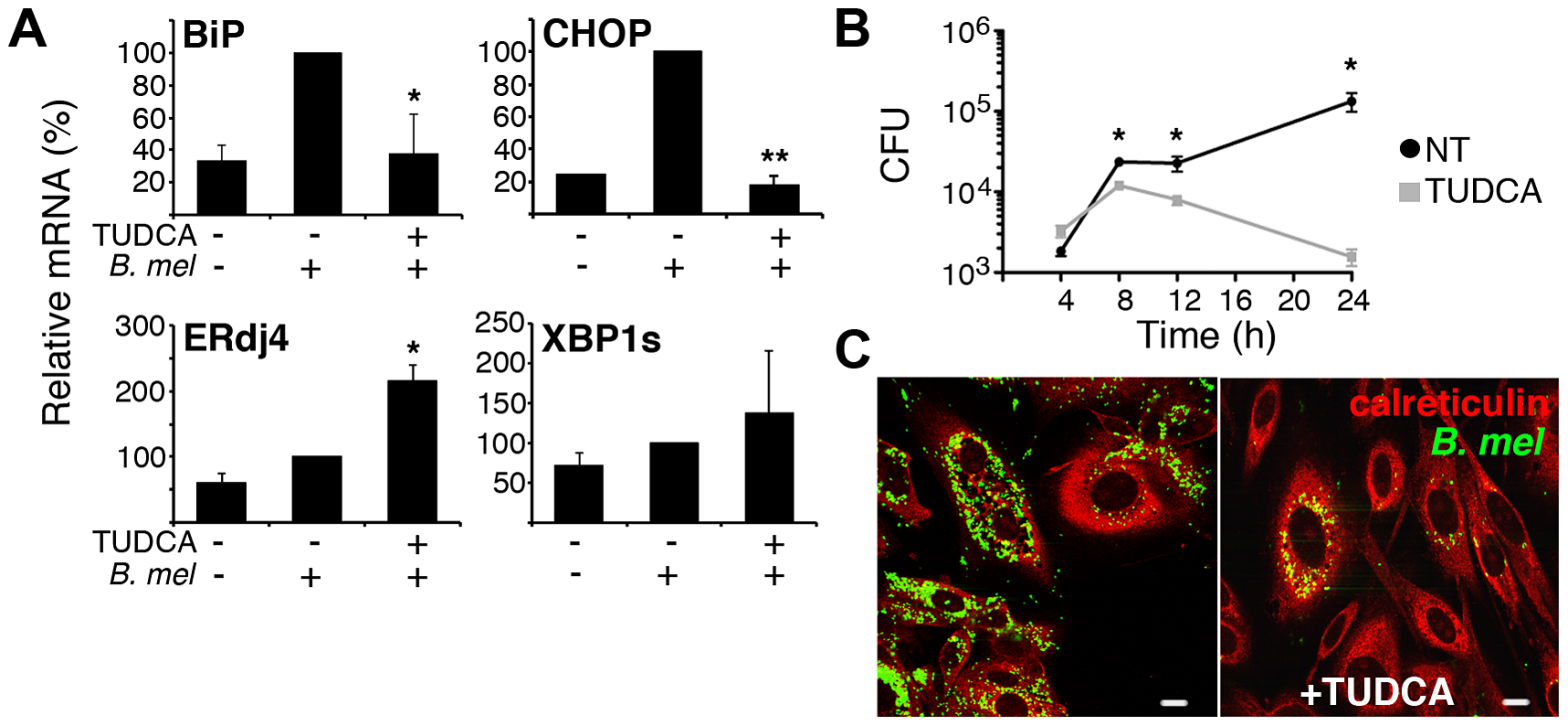
The chemical chaperone TUDCA inhibits BiP and CHOP induction and *Brucella* replication. A) RAW cells were pre-treated with 500 µg/mL TUDCA, and then infected with 100 MOI *Brucella* (*B. mel*) for 30 min., washed, and incubated 24 h prior to harvesting for RNA analysis by qPCR. Results were combined from 3–4 independent experiments by normalization to *B. melitensis* induced UPR gene expression ( = 100%), *p≤0.04 and p = 0.00003 vs. *B. melitensis* infection and NS vs. uninfected. For XBP1s and ERdj4, N = 2 independent experiments. B) RAW cells were not treated (black circles) or pre-treated with TUDCA (gray squares), infected as in (A), and lysed at different times following infection. CFU (colony forming units) were determined by transfer to dilution plates. *P≤0.04. Similar results were obtained with 10 MOI *Brucella* (not shown). Results are representative of 6 independent experiments. C) D17 osteosarcoma cells were untreated (left) or pre-treated with 500 µg/mL TUDCA (right), infected with *Brucella*-YFP (green) for 24 h, fixed, and stained with anti-calreticulin (red).

The pleiotropic effects of TcpB within an individual cell may have multiple effects on initial uptake, early bacterial destruction, trafficking, replication and ultimate recoverable CFU. For instance, the cytoskeletal disruption and potential alteration of vesicular trafficking induced by TcpB could initially impede *Brucella* infection. This interpretation is consistent with the increased initial CFU observed in the TcpB mutant cultures. An initial negative effect of TcpB on invasion could obscure a later positive effect on replication (and thus detected CFU). As a separate issue, although TcpB may play a role in inducing the UPR, other molecules may compensate in the absence of TcpB, as evident by the residual CHOP induction (Figure 5) and another recent report implicating VceC [40]. To more directly assess the role of the UPR in *Brucella* replication in macrophages, the cells were treated with TUDCA to inhibit the UPR. We confirmed that TUDCA pre-treatment decreases *Brucella* induced BiP and CHOP expression at 24 h (Figure 10). The effect of TUDCA on XBP1 splicing was variable (similar to the effect on tunicamycin-induced UPR), and ERdj4 expression increased. During a 24 h period, TUDCA had minimal impact on RAW cell viability (93±10% untreated) and none on *Brucella* at 500 µg/mL (Figure S6). At earlier time points (12–16 h), the effect of TUDCA on replication was modest but reproducible (5.4±1.8 fold mean decrease for 4 experiments). However, TUDCA pre-treatment significantly decreased recoverable *Brucella* CFU, typically by a log or more (p≤0.02 in 4 independent experiments, range 4-fold to 3 logs) by 24–36 h. TUDCA exerted a similar effect on *Brucella* CFU in the osteosarcoma D17 cell line (Figure S7A) [37], [38]. TUDCA did not appear to inhibit *Brucella* trafficking to a peri-nuclear location in those cells containing visible bacteria. Together, these data are consistent with a critical role for UPR pathways in enabling *Brucella* intracellular replication inside macrophages.

## Discussion

*B. melitensis* infection mobilizes all three UPR signaling axes in macrophages, stemming from the activation of IRE1, PERK and ATF6. Oxidative stress also strongly activates the PERK pathway, thus the UPR is often referred to as an “integrated stress response” [41]. However robust induction of target genes from the three distinct biochemical signaling pathways is most consistent with the UPR [42]. One report demonstrated IRE1 phosphorylation and PERK pathway activation in *M. tuberculosis* infected macrophages *in vivo*. However the direct link between infection and induction of host UPR was not established [19]. Another study implicated the IRE1 pathway in supporting *Brucella* replication, consistent with the results obtained in this study, however the relevance to macrophages was unclear [13]. Although previous data reported XBP1 mRNA splicing by intracellular bacteria such as *Francisella* [20], this is one of the first reports of more widespread UPR induction resulting directly from intracellular bacterial infection rather than toxin production.

*Brucella* induced XBP1 mRNA splicing appears to proceed predominantly through the previously described MyD88 (TLR) dependent pathway [20]. The unusual smooth *Brucella* LPS contains reduced negative charges and unusually long aliphatic hydrocarbon chains in the Lipid A core (C28 as compared to C12-16 in enterobacteria) [4]. Related to these properties, *Brucella* smooth LPS displays reduced TLR4 agonist activity [43]. Thus smooth LPS may be a relatively weak inducer of XBP1 splicing. Consistent with this prediction, the genomic island 2 deletion *Brucella* mutant that expresses rough LPS triggers much more robust XBP1 splicing (data not shown) [44]. In comparison with XBP1 splicing, downstream CHOP and ERdj4 target gene induction was entirely MyD88-independent. BiP induction was not detected in these particular experiments, potentially related to timing (BiP upregulation is an early transient event), mouse strain, or differences in macrophage type [45]. Thus, as noted by others, all signaling pathways encompassed by the UPR are not always coordinately regulated [20]. “UPR” signaling events such as XBP1 splicing may be triggered by non-UPR agonists and UPR signaling pathways are not always activated in their entirety. Other examples of XBP1 splicing-downstream target disconnection come from the viral literature, and Hepatitis C in particular [46]. The mechanism underlying the dissociation remains unknown. In the present study, it was curious that the canonical XBP1 target gene ERdj4 was up regulated in the absence of significantly detectable XBP1 splicing in the MyD88−/− bone marrow macrophages. There are several possible explanations. First, sufficient TLR4-TRIF dependent XBP1 activity remains to induce ERdj4. Second, only part of XBP1 splicing is MyD88 dependent and the assay is not sufficiently sensitive to detect minor differences. The low level XBP1 splicing induced by purified TcpB is consistent with this idea, as we would assume this is ER stress rather than MyD88-related. Indeed, TcpB would be expected to antagonize TLR-MyD88-dependent signaling [29], [30]. Third, ERdj4 may be induced in an XBP1 independent manner [20], [47]. Given the evidence that even weak TLR signaling by *Brucella* still induces XBP1 splicing, it will be interesting to determine the role of XBP1 in *Brucella*-induced cytokine production.

Our results reveal a new role for the TcpB protein in regulating host stress responses and ER structure. Indeed, the ability of TcpB to fragment and condense the ER may be the underlying mechanism for the dramatic ER restructuring first reported almost a decade ago [7]. Based on 1) our data correlating ER disruption and UPR induction in response to purified TcpB, 2) the diminished UPR and ER structural impact in the absence of TcpB, and 3) the capacity of an analogous microtubule disrupting drug paclitaxel to induce ER stress, it is highly likely that TcpB induced ER restructuring and UPR are causally linked [33], [34]. The comparison with tunicamycin treatment and the lack of TUDCA effect on TcpB induced ER restructuring also suggest that TcpB-induced UPR occurs following, or downstream of ER structural disruption. However, it remains possible that ER stress is not directly related to TcpB-induced ER structural changes. Purified TcpB was more effective at upregulating CHOP and BiP than IRE1 dependent events such as XBP1 splicing and ERdj4. Indeed XBP1 splicing was intact in the TcpB mutant infected cells and minimally induced in cells by TcpB protein. The vast majority of XBP1 splicing appears to proceed through the TLR-MyD88 pathway. Thus the readout of “UPR” reflects contributions from multiple bacterial factors. The effect of TUDCA on replication also suggest that the delayed virulence of the TcpB mutant in a susceptible IRF1−/− deficient mouse model may reflect both altered replication and enhanced cytokine production. It is a testament to bacterial efficiency that one protein product may both antagonize host immune signaling and induce host stress responses that support bacterial replication.

The ultimate role of TcpB in replication, given the pleiotropic effects of this molecule, remains unclear. Initially, uptake of the mutant is much greater in macrophages, but the growth curve plateaus below the level of the wild type. Growth of the ΔBtp1 *B. abortus* was not impaired in dendritic cells [29]. This may reflect timing, a difference in macrophages vs. dendritic cells, cell line vs. primary cells on another strain background, or differences in *B. abortus* vs. *B. melitensis*. The TcpB mutant is clearly attenuated *in vivo*, though immune modulation complicates the interpretation [30].

Although TcpB appears to play a pivotal role in regulating UPR target genes, other virulence factors (e.g. VirB) may contribute. Indeed, CHOP expression was not reduced to the non-infected level in the TcpB mutant infection, consistent with the existence of other UPR inducing molecules [40]. Also, TUDCA inhibited growth of the TcpB mutant (Figure S7B). The experimental variability obtained with the VirB mutants may reflect a timing issue (important earlier or later than our experimental window) or sensitivity. The proportion of cells infected and number of bacteria/cell will affect UPR detection. Since the VirB mutant traffics abnormally and fails to survive inside macrophages, fewer bacteria will be available to produce UPR-inducing factors [7]. Interestingly, in the IRF1−/− mouse model, patterns of *in vivo* virulence differed between the VirB and TcpB mutants consistent with roles in different parts of the bacterial life cycle. TcpB appears to regulate early spread of infection whereas VirB contributes more to bacterial persistence [28], [30]. The requirement for living bacteria to optimize UPR target gene induction suggests the UPR is an active process supported by new protein(s) or other factor(s) produced following infection; it is not just a host response to components present in dead bacteria. The residual UPR induction by heat-killed bacteria may reflect TcpB, or an unidentified factor produced by the bacteria during growth in broth.

The UPR may support the intracellular life cycle of *Brucella* in a number of ways. First, the UPR mobilizes amino acid transport and supports lipid biogenesis. Second, the UPR also initiates autophagy, thus providing more nutrients. As described by Starr et al, the UPR regulated autophagy may participate in completing the *Brucella* intracellular life cycle, allowing spread to neighboring cells [17]. Third, the UPR enhances protein-folding capacity through induction of chaperones and other folding machinery. Fourth, the UPR allows cells to cope with oxidative stresses. Finally, as a means of physiological adaptation, the UPR may enable host cells to survive the disruption of ER structure and function. The UPR encompasses anti-apoptotic mechanisms and only promotes apoptosis when stress is severe or prolonged. In the viral literature, Dengue activates all three UPR pathways, yet suppresses downstream apoptosis [48]. It will be interesting to determine if some of the same apoptosis modulating mechanisms apply to *Brucella*. Another possibility is that *Brucella* LPS may sufficiently temper CHOP induction to avert apoptosis [20].

In this study, TUDCA pre-treatment exerted a dramatic effect, decreasing recoverable CFU in culture. The simplest interpretation is that the host UPR plays an absolutely critical role in supporting *Brucella* replication. This hypothesis is consistent with the work from Qin et al. showing decreased *Brucella* CFU following IRE-1 knockdown. We also have preliminary data suggesting this UPR axis supports replication in macrophages, most likely through the IRE1-kinase-JNK signaling pathway rather than through XBP1 (data not shown). The contrasting effect of TUDCA on *Brucella* replication and XBP1 splicing/ERdj4 expression is consistent with our preliminary XBP1 RNAi data showing no effect on replication. The XBP1 variability in response to TUDCA may reflect multiple mechanisms of XBP1 splicing induction. However the apparent effect of TUDCA on *Brucella*-induced BiP and CHOP expression may also result from greatly diminished numbers of bacteria. Although TUDCA is widely utilized to assess the role of the UPR *in vivo*, and is approved for use in humans, the drug may affect other cellular processes besides the UPR [49]. The non-specificity of TUDCA is one limitation of this study. However, these results supply strong rationale to further investigate which specific UPR-related molecules might be involved in supporting *Brucella* replication in macrophages. Also, despite non-specificity, TUDCA may be useful therapeutically, particularly in view of safety and cost. It may prove important to inhibit multiple arms of the UPR, as inhibition of one specific signaling axis may not be sufficient. Successful inhibition of *Brucella* virulence *in vivo* by TUDCA or other more selective UPR modulation would open a new avenue of drug development.

TUDCA has an excellent safety profile and is being studied in humans to counteract UPR-related metabolic syndromes [49], [50]. It will be essential to determine whether TUDCA mediated inhibition of replication outweighs the effect of UPR blockade on inflammatory cytokine production *in vivo*. We, along with other researchers, have described dramatic augmentation of interferon and inflammatory cytokine production by the UPR [20], [51]. Indeed, the UPR has been implicated in numerous inflammatory and autoimmune diseases [52], [53]. Currently, little is known about the role of the UPR in immune responses to *Brucella*, and the formation of immune memory [54].

The concept that subverting the host UPR enables bacterial replication in macrophages, thus promoting infectious success represents a paradigm shift for the field that merits further investigation. The results from this study have broad implications for other bacteria that establish an intracellular replicative niche, particularly those that interact with the ER [55].

## Materials and Methods

### Cells, bacterial strains, and reagents

The RAW264.7 murine macrophage and D17 canine osteogenic sarcoma cell lines (both ATCC) were maintained in RPMI 1640/high glucose with 4 mM L-glutamine, sodium pyruvate (Hyclone Laboratories) and supplemented with 10% FBS (Hyclone), 100 U/mL penicillin, and 100 µg/mL streptomycin. *B. melitensis* 16M, the engineered bioluminescent strain GR019 (VirB mutant), or the TcpB deletion mutant were grown in *Brucella* broth (BB, Difco) supplemented with 50 µg/mL kanamycin [28], [30]. To heat kill bacteria, *B. melitensis* in BB was incubated at 65°C for 60 min. The purification of TcpB protein has been described [30]. MBP-TcpB was used at a concentration of 50 µg/mL, with maltose binding protein (MBP) as a control [30].

### Mice and bone marrow derivation

Mice were kept in facilities at the University of Wisconsin-Madison that are accredited by the American Association of Laboratory Animal Care. Mouse experiments were performed with oversight and approval of the University of Wisconsin-Madison School of Medicine and Public Health and School of Veterinary Medicine Animal Care and Use Committee (NIH assurance number: A3368-01), in accordance with recommendations in the Guide for the Care and Use of Laboratory Animals of the National Institutes of Health. MyD88−/− femurs were a gift from Laura Knoll, University of Wisconsin-Madison. Bone marrow cells from C57BL/6 wild type or MyD88−/− femurs were isolated on Histopaque 1083 (Sigma-Aldrich, St. Louis, MO) and differentiated for 7 days in RPMI 1640 with 10% FBS and 50 ng/mL recombinant murine M-CSF (Peprotech). For *in vivo* infections, 4–7 mice/group of 6–8 week old BALB/c mice were injected i.p. with PBS or 10^7^ GR023 (bioluminescent *B. melitensis*). After 24 h, spleens were pooled within groups, homogenized, and splenic macrophages were isolated using CD11b+ magnetic cell separation (Miltenyi) according to manufacturer's protocol. Cells were immediately resuspended in Trizol for further processing.

### *In vitro* infections

RAW 264.7 or bone marrow derived macrophages (BMDM) were cultured in 6-well dishes (unless otherwise indicated) overnight prior to infection. Macrophages were infected with either 10∶1 or 100∶1 multiplicity of infection (MOI) with late log or stationary phase *Brucella* for times indicated and then harvested for RNA analysis or CFU evaluation. Cultures were incubated at 37°C with 5% CO_2_. Select cultures were treated with 0.01 µg/mL tunicamycin (Sigma) 30 minutes prior to infection. Note: although gentamycin is routinely used during *Brucella* infections, it decreases detection of UPR induction (particularly CHOP).

### Complementation assay

The coding sequence of Bmel 1674 encoding TcpB1 was inserted into the *Brucella* plasmid pNstrcD [56]. The plasmid was electroporated into *B. melitensis* ΔTcpB1 by standard methods. RAW 264.7 or J774A.1 (both from ATCC) mouse macrophage cell lines were seeded in 6 well tissue culture plates at 3×10^5^ per well 1 day prior to infection. Cultures of *B. melitensis*, *B. melitensis* ΔTcpB1, and *B. melitensis* ΔTcpB1+pNstrcD/BmeI1674 (3 ml each in BHI media with appropriate antibiotics) were seeded 2–3 days before infection to be in late log phase at the time of infection. Macrophage cells were infected at 100 MOI and cultured for 24 h. Cells were then washed 1× in PBS, lysed and harvested in 1 ml/well of Trizol (Invitrogen) for RNA processing.

### UPR detection (PCR)

Real time PCR: Following culture, supernatant was removed and samples were resuspended in TRIzol (Invitrogen). RNA was purified according to manufacturer's instructions and treated with DNaseI (Invitrogen) to remove genomic DNA. RNA was reverse transcribed using random primers (Promega). Relative cDNA was quantified using SYBR Green (Bio-Rad) and detection in MyiQ, or CFX96 real time PCR machines (both Bio-Rad). Primers were designed using Beacon Design software (Premier Biosoft) and are as follows: 18S rRNA: forward, 5′-GGACACGGACAGGATTGACAG-3′ and reverse, 5′-ATCGCTCCACCAACTAAGAACG-3′. Hprt1: forward, 5′-GTTAAGCAGTACAGCCCCCAAA-3′ and reverse, 5′-AGGGCATATCCAACAACAAACTT. BiP: forward, 5′-AGGATGCGGACATTGAAGAC-3′ and reverse, 5′-AGGTGAAGATTCCAATTACATTCG-3′. CHOP: forward, 5′-CATCACCTCCTGTCTGTCTC-3′ and reverse, 5′-AGCCCTCTCCTGGTCTAC-3′. ERdj4: forward, 5′-AGGGAAGGATGAGGAAATCG-3′ and reverse, 5′-ACTGTTGTTGCCGTTTGG-3′. IL-6: forward, 5′-ACGATGATGCACTTGCAGA-3′ and reverse, 5′-GTAGCTATGGTACTCCAGAAGAC-3′. XBP1 splicing was assessed through 3 assays: 1) Agarose gel assay: XBP-1 primers for conventional PCR: forward, 5′-ACACGCTTGGG- AATGGACAC-3′ and reverse, 5′-CCATGGGAAGATGTTCTGGG-3′. PCR amplified cDNA was resolved on 3% gel and optical density (OD) quantified using Image Quant (GE Healthcare). % XBP splicing is spliced cDNA OD/total (spliced+unspliced) OD×100. 2) Quantification of separate species by Agilent. 3) qPCR assay: XBP1(t): forward, 5′-TCCGCAGCACTCAGACTATGT-3′ and reverse, 5′-ATGCCCAAAAGGATATCAGACTC-3′. XBP1(s): forward, 5′-GAGTCCGCAGCAGGTG-3′ and reverse, 5′-GTGTCAGAGTCCATGGGA-3′. % splicing = XBP1(s)/XBP1(t)×100 [57].

### UPR detection (biochemistry)

Non-infected and *B.melitensis* infected RAW cells were harvested and resuspended in a buffer containing 20 mM Tris-HCI [8.0] and 0.5% SDS. Samples were boiled for 20 min. and mixed with equal amount of sample buffer. Cell lysates were resolved on a 4–20% SDS PAGE and transferred to immobilon PVDF membrane (Millipore). The membrane was blocked with Tris-buffered saline containing 0.1% Tween 20 (TTBS) and 5% nonfat milk for 1 h at room temperature followed by three washes with TTBS. The membrane was incubated with anti-CHOP antibody (Cell Signaling Technology) in blocking buffer over night at 4°C. After washing three times with TTBS, the membrane was incubated with HRP-conjugated anti-mouse IgG (Pierce) in blocking buffer for 1 h at room temperature. After three washes with TTBS, protein bands were detected using SuperSignal West Pico Chemiluminescent Substrate according to manufacturer's instructions (Pierce). The membrane was re-probed with anti-actin (Santa Cruz Biotechnology). Chemiluminescence was detected by CL-XPosure Film (Thermo Scientific).

### Immunofluorescence microscopy

RAW264.7 cells, D17 cells or bone marrow derived macrophages were seeded into chamber slides (Lab Tek and Ibidi) and allowed to adhere 16–24 h. For infections, macrophages were infected (1000 MOI) with either wild-type *B. melitensis* or *B. melitensis* containing a TcpB gene deletion for 24 h. Both strains express YFP under control of the trcD promoter. TUDCA (500 µg/mL) pre-treatments were 30–60 min. For purified protein treatments, the medium was then replaced with fresh medium (1 ml) containing purified maltose binding protein (MBP), MBP-TcpB protein (10 or 50 µg/ml), or tunicamycin (1 or 10 µg/mL) and the plates were incubated over night (12–24 h). The cells were washed 3× with PBS, fixed with 4% paraformaldehyde for 10 min, followed by permeabilization with 0.1% Triton X100 for 10 min. Cells were treated with blocking buffer containing 5% normal serum and 50 mM NH_4_CI in 1X PBS for 30 min, then washed and incubated with 1∶100 dilution of anti-calreticulin antibody (Thermo Scientific) in PBS containing 0.1% normal serum for 1 h. Cells were washed 3X with PBS and incubated with 1∶1000 dilution of Alexa Fluor 488 goat anti-rabbit IgG (Invitrogen) or secondary conjugated to DyLight 550 (Thermo Scientific) for 1 h, or anti-rabbit 550 (Cell Signaling) overnight, washed 3X with PBS, and mounted in *ProLong Gold* antifade reagent (Invitrogen). Select samples were mounted in *ProLong Gold* antifade reagent with DAPI (Cell Signaling). Images were collected using either a Radiance 2100 MP Rainbow confocal/multiphoton microscope (Bio-Rad) or Nikon A1R confocal laser microscope.

### UPR blockade

To determine the effect of chemical chaperones on *Brucella* viability, *Brucella* were plated in 96 well dishes at 5×10^6^ cells/well in RPMI containing serial dilutions of tauroursodeoxycholic acid (TUDCA, Sigma). BacTiter-Glo assay (Promega) was performed to determine ATP content (viability) as assessed by luminescence. To determine the effect on RAW cell viability, cells were plated in 96 well plates at 10^4^ cells/well one day prior to challenge. The medium was then replaced with fresh medium containing serial dilutions of chemical chaperones. CellTiter-Glo assay (Promega) was performed to determine ATP content (viability) as detected by luminescence. Effect of TUDCA on *Brucella* viability in broth was also determined utilizing this assay. To confirm TUDCA inhibition of UPR, RAW cells were pre-treated 30 min. with TUDCA, then stimulated with 10 µg/mL tunicamycin (Sigma) for 6 h or *B. melitensis* for 24 h prior to processing in TRIzol. Inhibition of replication: RAW 264.7 macrophages were plated in 24 well dishes at 5×10^5^/well the day prior to infection. Cells were pre-treated with 500 µg/ml TUDCA (4 experiments) or 4 mg/mL TUDCA (1 experiment, no significant RAW cell viability effect) for 30 min. prior to infection with either 10 or 100 MOI of stationary phase *B. melitensis*. After 30 min., cells were washed 4X with warm PBS and fresh media with 50 µg/ml gentamycin added with or without TUDCA. To evaluate colony-forming units (CFU), cells were washed 3X with PBS and then lysed in 1% Triton-X 100 in water. CFU were determined by serial dilution plating on agar after 3–4 days. In parallel, samples were lysed in TRIzol to determine effect of TUDCA on UPR target gene induction at 24 h.

### Statistical analysis

Differences between data were evaluated using Students T-test with p<0.05 considered significant.

## Supporting Information

Figure S1 **TcpB co-localizes with ER calreticulin.** D17 cells were transfected with pCMV-TcpB-HA plasmid and fixed 24 h later [31]. Cells were stained with anti-HA (red), anti-calreticulin (green) and DAPI (blue), and imaged by fluorescence microscopy (50X). Co-localization of TcpB-HA and calreticulin appears yellow. Similar results were obtained in RAW 264.7 cells. Images are 50X.
(TIF)

Figure S2 **TcpB mutant** ***Brucella*** **infection induces less ER structural disruption.** RAW 264.7 cells were infected with an YFP-expressing TcpB deletion mutant (ΔTcpB) or wild type (WT) *B. melitensis* (green) for 24 h (as in Figure 6). The ER is visualized with anti-calreticulin (red). Broad field images from 2 experiments are shown.
(TIF)

Figure S3 **Comparison of untreated, MBP-treated and TcpB treated cell ER structure.** RAW cells were treated with 50 µg/mL purified MBP or MPB-TcpB for 12 h. The ER is visualized with anti-calreticulin (red). Arrow indicates ER condensation and fragmentation. Bar is 20 µM.
(TIF)

Figure S4 **TcpB increases vacuole diameter and ER size.** RAW 264.7 cells were cultured in the presence of 50 µg/ml purified TcpB for 24 hours then washed and fixed for staining as shown in Figures 7–8. Five non-dividing cells in each frame were randomly selected for quantification. Measurements were taken using the Ruler Tool within Adobe Photoshop, with the scale set at 1 pixel = 62.15 nm for 100× magnified microscopy images. A) Vacuole diameters were measured in control (29 vacuoles) and TcpB treated (47 vacuoles) cells. TcpB treatment increased vacuole diameter significantly (***P≤0.0003). B) Calreticulin staining area was assessed by measuring the entire anti-calreticulin labeled fluorescent area and then subtracting out the area of the nuclei. Calreticulin area was significantly increased in TcpB treated cells (*P≤0.03), while nuclear size was equivalent to the control cells (data not shown).
(TIF)

Figure S5 **TUDCA inhibits cytokine induction by the ER stressor tunicamycin.** RAW264.7 macrophages were pre-treated with 500 µg/mL TUDCA 30 min., followed by 6 h 10 µg/mL tunicamycin (Tm) as indicated, and then harvested for RNA. Relative cytokine gene expression was assessed by qPCR. Results are combined from 2 independent experiments.
(TIF)

Figure S6 **Minimal effects of TUDCA on host cell and pathogen viability.** A) left panel: RAW 264.7 macrophages were treated with 500 µg/mL for the times indicated. Viability (ATP content) was determined by Cell-titer glo assay. Error bars represent standard deviation of duplicate (non-treated control, black diamonds) and triplicate (TUDCA, gray squares) determinations. Results are from an experiment showing the greatest effect of TUDCA out of 4 independent experiments. *p = 0.04. Right panel: Bars depict average of 4 experiments at the 24 h time point, normalized to untreated control (set = 1.0). B) *B. melitensis* in broth culture were untreated (NT) or treated with TUDCA as above for times indicated and ATP content determined by Cell-titer glo assay.
(TIF)

Figure S7 **TUDCA inhibits** ***Brucella*** **replication in both RAW264.7 macrophages and D17 cells.** A) RAW cells (blue) or D17 cells (red) were not treated (solid) or pre-treated with 500 µg/mL TUDCA (striped) for 30 minutes, infected with 100 MOI of *B. melitensis*, and lysed at 16 h following infection. CFU (colony forming units) were determined by transfer to dilution plates. Error bars are standard deviation of triplicate determinations. *P = 0.02. B) RAW cells were untreated (black circles) or pre-treated with 500 µg/mL TUDCA as above (gray squares), and then infected with 10 MOI of the ΔTcpB mutant *Brucella*. CFU were determined as in (A). Error bars represent standard deviation of 4 determinations, *p<0.04, **p<0.006.
(TIF)

## References

[1] Pappas G, Akritidis N, Bosilkovski M, Tsianos E (2005) Brucellosis. N Engl J Med 352: 2325–2336. 15930423 10.1056/NEJMra050570

[2] Pappas G, Papadimitriou P, Akritidis N, Christou L, Tsianos EV (2006) The new global map of human brucellosis. Lancet Infect Dis 6: 91–99. 16439329 10.1016/S1473-3099(06)70382-6

[3] Atluri VL, Xavier MN, de Jong MF, den Hartigh AB, Tsolis RE (2011) Interactions of the human pathogenic Brucella species with their hosts. Annu Rev of Microbiol 65: 523–541. 21939378 10.1146/annurev-micro-090110-102905 PMC13363517

[4] Lapaque N, Moriyon I, Moreno E, Gorvel JP (2005) Brucella lipopolysaccharide acts as a virulence factor. Curr Opin Microbiol 8: 60–66. 15694858 10.1016/j.mib.2004.12.003

[5] Martirosyan A, Moreno E, Gorvel JP (2011) An evolutionary strategy for a stealthy intracellular Brucella pathogen. Immunol Rev 240: 211–234. 21349096 10.1111/j.1600-065X.2010.00982.x

[6] Kim S, Watarai M, Suzuki H, Makino S, Kodama T, et al (2004) Lipid raft microdomains mediate class A scavenger receptor-dependent infection of Brucella abortus. Microb Pathog 37: 11–19. 15194155 10.1016/j.micpath.2004.04.002

[7] Celli J, de Chastellier C, Franchini DM, Pizarro-Cerda J, Moreno E, et al (2003) Brucella evades macrophage killing via VirB-dependent sustained interactions with the endoplasmic reticulum. J Exp Med 198: 545–556. 12925673 10.1084/jem.20030088 PMC2194179

[8] Fugier E, Salcedo SP, de Chastellier C, Pophillat M, Muller A, et al (2009) The glyceraldehyde-3-phosphate dehydrogenase and the small GTPase Rab 2 are crucial for Brucella replication. PLoS Pathog 5: e1000487. 19557163 10.1371/journal.ppat.1000487 PMC2695806

[9] Celli J, Salcedo SP, Gorvel JP (2005) Brucella coopts the small GTPase Sar1 for intracellular replication. Proc Natl Acad Sci U S A 102: 1673–1678. 15632218 10.1073/pnas.0406873102 PMC547823

[10] Boschiroli ML, Ouahrani-Bettache S, Foulongne V, Michaux-Charachon S, Bourg G, et al (2002) The Brucella suis virB operon is induced intracellularly in macrophages. Proc Natl Acad Sci U S A 99: 1544–1549. 11830669 10.1073/pnas.032514299 PMC122227

[11] Schroder M, Kaufman RJ (2005) The mammalian unfolded protein response. Annu Rev Biochem 74: 739–789. 15952902 10.1146/annurev.biochem.73.011303.074134

[12] Hetz C, Glimcher LH (2009) Fine-tuning of the unfolded protein response: Assembling the IRE1alpha interactome. Mol Cell 35: 551–561. 19748352 10.1016/j.molcel.2009.08.021 PMC3101568

[13] Qin QM, Pei J, Ancona V, Shaw BD, Ficht TA, et al (2008) RNAi screen of endoplasmic reticulum-associated host factors reveals a role for IRE1alpha in supporting Brucella replication. PLoS Pathog 4: e1000110. 18654626 10.1371/journal.ppat.1000110 PMC2453327

[14] Ogata M, Hino S, Saito A, Morikawa K, Kondo S, et al (2006) Autophagy is activated for cell survival after endoplasmic reticulum stress. Mol Cell Biol 26: 9220–9231. 17030611 10.1128/MCB.01453-06 PMC1698520

[15] Pizarro-Cerda J, Meresse S, Parton RG, van der Goot G, Sola-Landa A, et al (1998) Brucella abortus transits through the autophagic pathway and replicates in the endoplasmic reticulum of nonprofessional phagocytes. Infect Immun 66: 5711–5724. 9826346 10.1128/iai.66.12.5711-5724.1998 PMC108722

[16] Guo F, Zhang H, Chen C, Hu S, Wang Y, et al (2012) Autophagy favors Brucella melitensis survival in infected macrophages. Cell Mol Biol Lett 17: 249–57. 22367856 10.2478/s11658-012-0009-4 PMC6275789

[17] Starr T, Child R, Wehrly TD, Hansen B, Hwang S, et al (2012) Selective subversion of autophagy complexes facilitates completion of the Brucella intracellular cycle. Cell Host Microbe 11: 33–45. 22264511 10.1016/j.chom.2011.12.002 PMC3266535

[18] Pizarro-Cerda J, Moreno E, Sanguedolce V, Mege JL, Gorvel JP (1998) Virulent Brucella abortus prevents lysosome fusion and is distributed within autophagosome-like compartments. Infect Immun 66: 2387–2392. 9573138 10.1128/iai.66.5.2387-2392.1998 PMC108212

[19] Seimon TA, Kim MJ, Blumenthal A, Koo J, Ehrt S, et al (2010) Induction of ER stress in macrophages of tuberculosis granulomas. PLoS One 5: e12772. 20856677 10.1371/journal.pone.0012772 PMC2939897

[20] Martinon F, Chen X, Lee AH, Glimcher LH (2010) TLR activation of the transcription factor XBP1 regulates innate immune responses in macrophages. Nat Immunol 11: 411–8. 20351694 10.1038/ni.1857 PMC3113706

[21] Pillich H, Loose M, Zimmer KP, Chakraborty T (2012) Activation of the unfolded protein response by Listeria monocytogenes. Cell Microbiol 14: 949–964. 22321539 10.1111/j.1462-5822.2012.01769.x

[22] Harding HP, Novoa I, Zhang Y, Zeng H, Wek R, et al (2000) Regulated translation initiation controls stress-induced gene expression in mammalian cells. Mol Cell 6: 1099–1108. 11106749 10.1016/s1097-2765(00)00108-8

[23] Lee AH, Iwakoshi NN, Glimcher LH (2003) XBP-1 regulates a subset of endoplasmic reticulum resident chaperone genes in the unfolded protein response. Mol Cell Biol 23: 7448–7459. 14559994 10.1128/MCB.23.21.7448-7459.2003 PMC207643

[24] Yamamoto K, Sato T, Matsui T, Sato M, Okada T, et al (2007) Transcriptional induction of mammalian ER quality control proteins is mediated by single or combined action of ATF6alpha and XBP1. Dev Cell 13: 365–376. 17765680 10.1016/j.devcel.2007.07.018

[25] Calfon M, Zeng H, Urano F, Till JH, Hubbard SR, et al (2002) IRE1 couples endoplasmic reticulum load to secretory capacity by processing the XBP-1 mRNA. Nature 415: 92–96. 11780124 10.1038/415092a

[26] Woo CW, Cui D, Arellano J, Dorweiler B, Harding H, et al (2009) Adaptive suppression of the ATF4-CHOP branch of the unfolded protein response by toll-like receptor signalling. Nat Cell Biol 11: 1473–1480. 19855386 10.1038/ncb1996 PMC2787632

[27] Oliveira SC, de Almeida LA, Carvalho NB, Oliveira FS, Lacerda TL (2011) Update on the role of innate immune receptors during Brucella abortus infection. Vet Immunol Immunopathol 148: 129–35. 21700343 10.1016/j.vetimm.2011.05.036

[28] Rajashekara G, Glover DA, Krepps M, Splitter GA (2005) Temporal analysis of pathogenic events in virulent and avirulent Brucella melitensis infections. Cell Microbiol 7: 1459–1473. 16153245 10.1111/j.1462-5822.2005.00570.x

[29] Salcedo SP, Marchesini MI, Lelouard H, Fugier E, Jolly G, et al (2008) Brucella control of dendritic cell maturation is dependent on the TIR-containing protein Btp1. PLoS Pathog 4: e21. 18266466 10.1371/journal.ppat.0040021 PMC2233671

[30] Radhakrishnan GK, Yu Q, Harms JS, Splitter GA (2009) Brucella TIR Domain-containing Protein Mimics Properties of the Toll-like Receptor Adaptor Protein TIRAP. J Biol Chem 284: 9892–9898. 19196716 10.1074/jbc.M805458200 PMC2665112

[31] Radhakrishnan GK, Harms JS, Splitter GA (2011) Modulation of microtubule dynamics by a TIR domain protein from the intracellular pathogen Brucella melitensis. The Biochem J 439: 79–83. 21692747 10.1042/BJ20110577 PMC3513345

[32] Wozniak MJ, Bola B, Brownhill K, Yang YC, Levakova V, et al (2009) Role of kinesin-1 and cytoplasmic dynein in endoplasmic reticulum movement in VERO cells. J Cell Sci 122: 1979–1989. 19454478 10.1242/jcs.041962 PMC2723153

[33] Wang J, Yin Y, Hua H, Li M, Luo T, et al (2009) Blockade of GRP78 sensitizes breast cancer cells to microtubules-interfering agents that induce the unfolded protein response. J Cell Mol Med 13: 3888–3897. 19674193 10.1111/j.1582-4934.2009.00873.x PMC4516536

[34] Liao PC, Tan SK, Lieu CH, Jung HK (2008) Involvement of endoplasmic reticulum in paclitaxel-induced apoptosis. J Cell Biochem 104: 1509–1523. 18452161 10.1002/jcb.21730

[35] Webster DR (2002) Microtubules in cardiac toxicity and disease. Cardiovasc Toxicol 2: 75–89. 12271151 10.1385/ct:2:2:075

[36] Citterio C, Vichi A, Pacheco-Rodriguez G, Aponte AM, Moss J, et al (2008) Unfolded protein response and cell death after depletion of brefeldin A-inhibited guanine nucleotide-exchange protein GBF1. Proc Natl Acad Sci U S A 105: 2877–2882. 18287014 10.1073/pnas.0712224105 PMC2268553

[37] Scian R, Barrionuevo P, Fossati CA, Giambartolomei GH, Delpino MV (2012) Brucella abortus invasion of osteoblasts inhibits bone formation. Infect Immun 80: 2333–2345. 22547546 10.1128/IAI.00208-12 PMC3416452

[38] Leclerq S, Harms JS, Rosinha GM, Azevedo V, Oliveira SC (2002) Induction of a th1-type of immune response but not protective immunity by intramuscular DNA immunisation with Brucella abortus GroEL heat-shock gene. J MedMicrobiol 51: 20–26. 10.1099/0022-1317-51-1-20 11803949

[39] Arenas GN, Staskevich AS, Aballay A, Mayorga LS (2000) Intracellular trafficking of Brucella abortus in J774 macrophages. Infect Immun 68: 4255–4263. 10858243 10.1128/iai.68.7.4255-4263.2000 PMC101738

[40] de Jong MF, Starr T, Winter MG, den Hartigh AB, Child R, et al (2013) Sensing of Bacterial Type IV Secretion via the Unfolded Protein Response. MBio 4: e00418–12. 23422410 10.1128/mBio.00418-12 PMC3624511

[41] Harding HP, Zhang Y, Zeng H, Novoa I, Lu PD, et al (2003) An integrated stress response regulates amino acid metabolism and resistance to oxidative stress. Mol Cell 11: 619–633. 12667446 10.1016/s1097-2765(03)00105-9

[42] Murray JI, Whitfield ML, Trinklein ND, Myers RM, Brown PO, et al (2004) Diverse and specific gene expression responses to stresses in cultured human cells. Mol Biol Cell 15: 2361–2374. 15004229 10.1091/mbc.E03-11-0799 PMC404029

[43] Duenas AI, Orduna A, Crespo MS, Garcia-Rodriguez C (2004) Interaction of endotoxins with Toll-like receptor 4 correlates with their endotoxic potential and may explain the proinflammatory effect of Brucella spp. LPS. Int Immunol 16: 1467–1475. 15339879 10.1093/intimm/dxh148

[44] Rajashekara G, Covert J, Petersen E, Eskra L, Splitter G (2008) Genomic island 2 of Brucella melitensis is a major virulence determinant: functional analyses of genomic islands. J Bacteriol 190: 6243–6252. 18641138 10.1128/JB.00520-08 PMC2546784

[45] Emadali A, Nguyen DT, Rochon C, Tzimas GN, Metrakos PP, et al (2005) Distinct endoplasmic reticulum stress responses are triggered during human liver transplantation. J Pathol 207: 111–118. 15912576 10.1002/path.1798

[46] Tardif KD, Mori K, Kaufman RJ, Siddiqui A (2004) Hepatitis C virus suppresses the IRE1-XBP1 pathway of the unfolded protein response. J Biol Chem 279: 17158–17164. 14960590 10.1074/jbc.M312144200

[47] Heldens L, Hensen SM, Onnekink C, van Genesen ST, Dirks RP, et al (2011) An atypical unfolded protein response in heat shocked cells. PLoS One 6: e23512. 21853144 10.1371/journal.pone.0023512 PMC3154502

[48] Pena J, Harris E (2011) Dengue virus modulates the unfolded protein response in a time-dependent manner. J Biol Chem 286: 14226–14236. 21385877 10.1074/jbc.M111.222703 PMC3077624

[49] Engin F, Hotamisligil GS (2010) Restoring endoplasmic reticulum function by chemical chaperones: an emerging therapeutic approach for metabolic diseases. Diabetes Obes Metab 12 Suppl 2: 108–115. 21029307 10.1111/j.1463-1326.2010.01282.x

[50] Kars M, Yang L, Gregor MF, Mohammed BS, Pietka TA, et al (2010) Tauroursodeoxycholic Acid may improve liver and muscle but not adipose tissue insulin sensitivity in obese men and women. Diabetes 59: 1899–1905. 20522594 10.2337/db10-0308 PMC2911053

[51] Smith JA, Turner MJ, DeLay ML, Klenk EI, Sowders DP, et al (2008) Endoplasmic reticulum stress and the unfolded protein response are linked to synergistic IFN-beta induction via X-box binding protein 1. Eur J Immunol 38: 1194–1203. 18412159 10.1002/eji.200737882 PMC2838478

[52] Hasnain SZ, Lourie R, Das I, Chen AC, McGuckin MA (2012) The interplay between endoplasmic reticulum stress and inflammation. Immunol Cell Biol 90: 260–270. 22249202 10.1038/icb.2011.112 PMC7165805

[53] Wang S, Kaufman RJ (2012) The impact of the unfolded protein response on human disease. J Cell Biol 197: 857–67. 22733998 10.1083/jcb.201110131 PMC3384412

[54] Butler NS, Nolz JC, Harty JT (2011) Immunologic considerations for generating memory CD8 T cells through vaccination. Cell Microbiol 13: 925–933. 21501363 10.1111/j.1462-5822.2011.01594.x PMC3116979

[55] Roy CR, Salcedo SP, Gorvel JP (2006) Pathogen-endoplasmic-reticulum interactions: in through the out door. Nat Rev Immunol 6: 136–147. 16491138 10.1038/nri1775 PMC7097709

[56] Seleem MN, Jain N, Alqublan H, Vemulapalli R, Boyle SM (2008) Sriranganathan N (2008) Activity of native vs. synthetic promoters in Brucella. FEMS Microbiol Lett 288: 211–215. 18811654 10.1111/j.1574-6968.2008.01358.x

[57] Cai WF, Pritchard T, Florea S, Lam CK, Han P, et al (2012) Ablation of junctin or triadin is associated with increased cardiac injury following ischaemia/reperfusion. Cardiovasc Res 94: 333–341. 22411973 10.1093/cvr/cvs119 PMC3331615

