# Supplementary figures and images for: Brucella Induces an Unfolded Protein Response via TcpB That Supports Intracellular Replication in Macrophages
Source: PLoS Pathog. 2013 Dec 5;9(12):e1003785. doi: 10.1371/journal.ppat.1003785 (PMC3855547; doi:10.1371/journal.ppat.1003785)

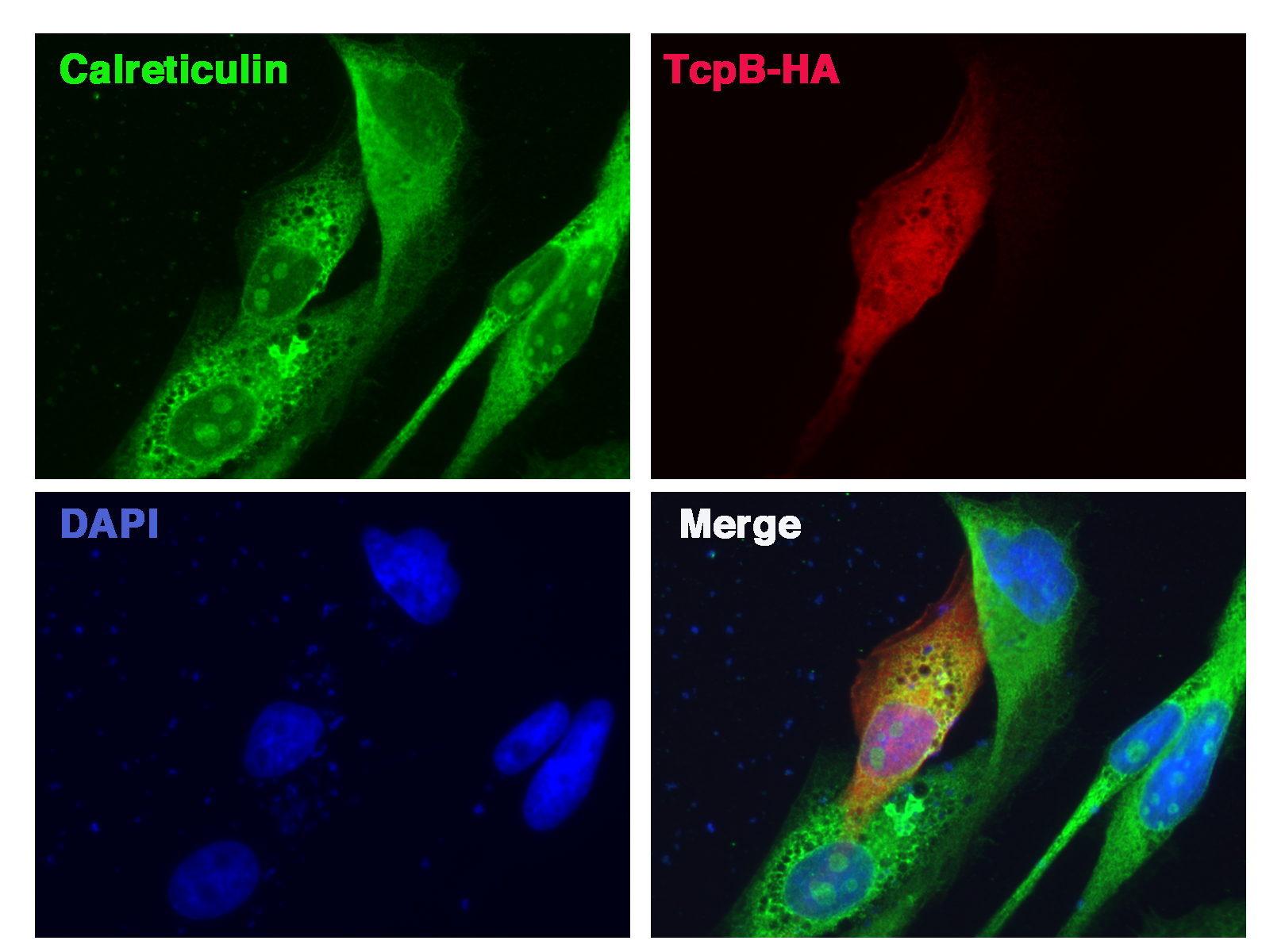

Supplement: Figure S1 — TcpB co-localizes with ER calreticulin. D17 cells were transfected with pCMV-TcpB-HA plasmid and fixed 24 h later [31]. Cells were stained with anti-HA (red), anti-calreticulin (green) and DAPI (blue), and imaged by fluorescence microscopy (50X). Co-localization of TcpB-HA and calreticulin appears yellow. Similar results were obtained in RAW 264.7 cells. Images are 50X. (TIF) [file ppat.1003785.s001.tif]

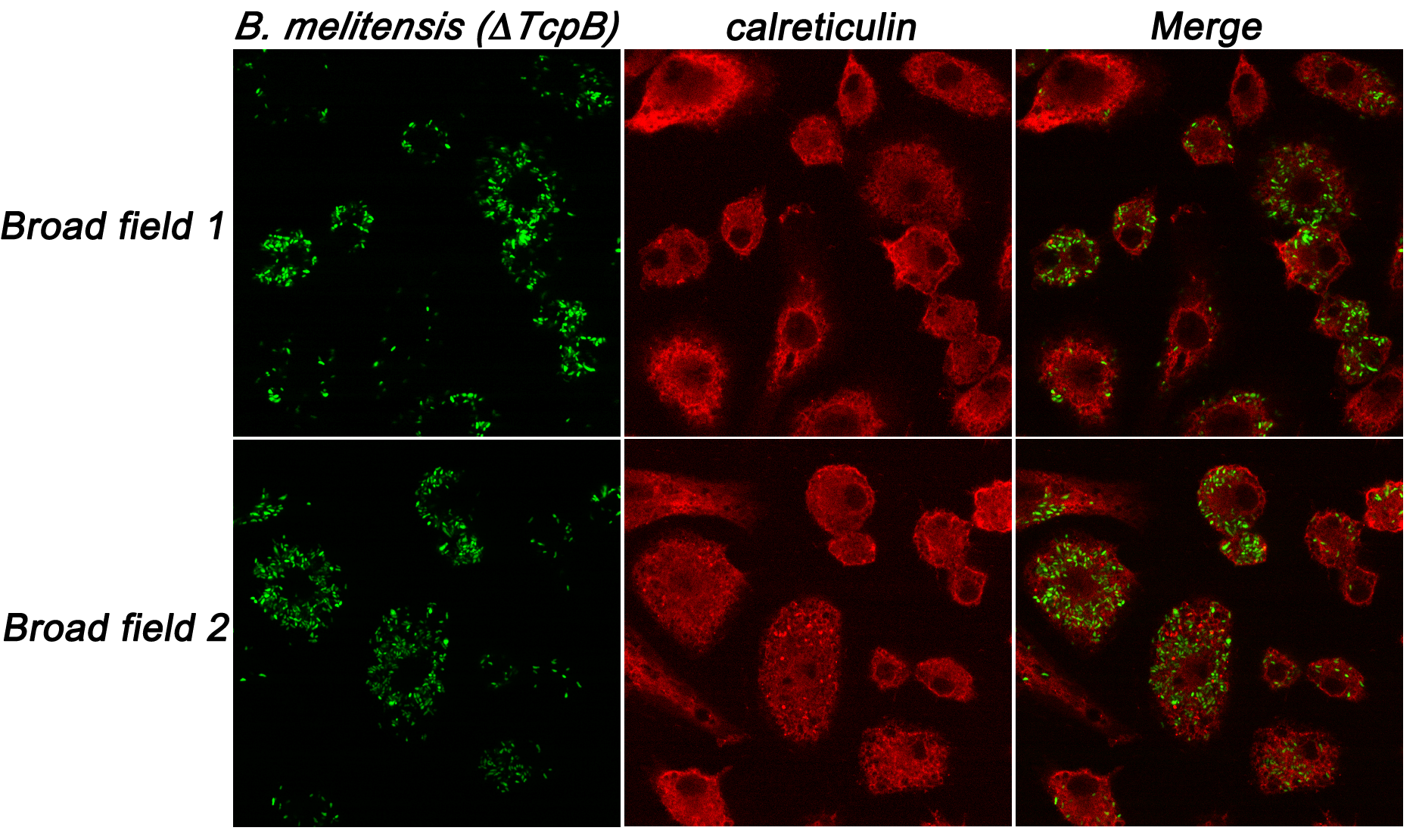

Supplement: Figure S2 — TcpB mutant Brucella infection induces less ER structural disruption. RAW 264.7 cells were infected with an YFP-expressing TcpB deletion mutant (ΔTcpB) or wild type (WT) B. melitensis (green) for 24 h (as in Figure 6). The ER is visualized with anti-calreticulin (red). Broad field images from 2 experiments are shown. (TIF) [file ppat.1003785.s002.tif]

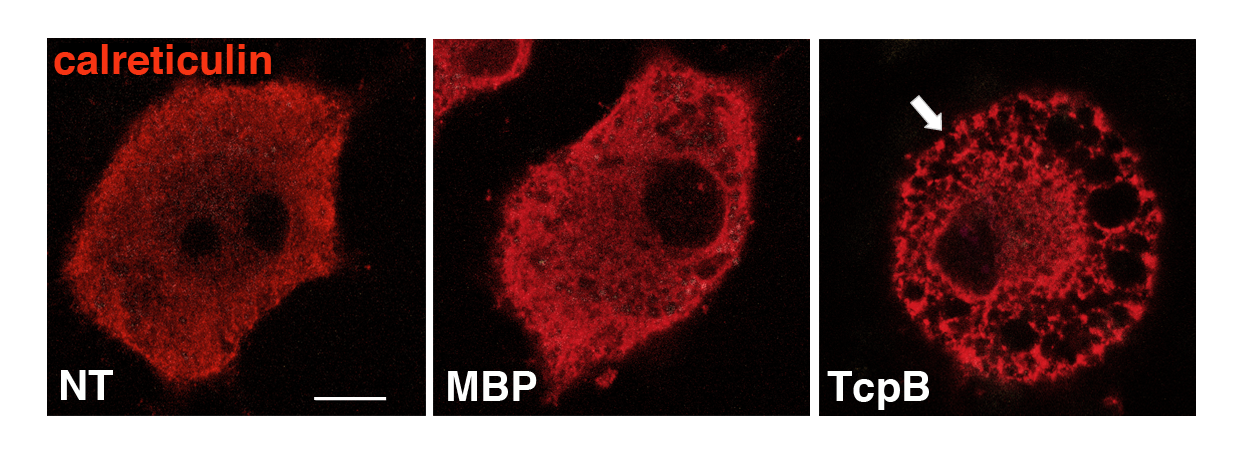

Supplement: Figure S3 — Comparison of untreated, MBP-treated and TcpB treated cell ER structure. RAW cells were treated with 50 µg/mL purified MBP or MPB-TcpB for 12 h. The ER is visualized with anti-calreticulin (red). Arrow indicates ER condensation and fragmentation. Bar is 20 µM. (TIF) [file ppat.1003785.s003.tif]

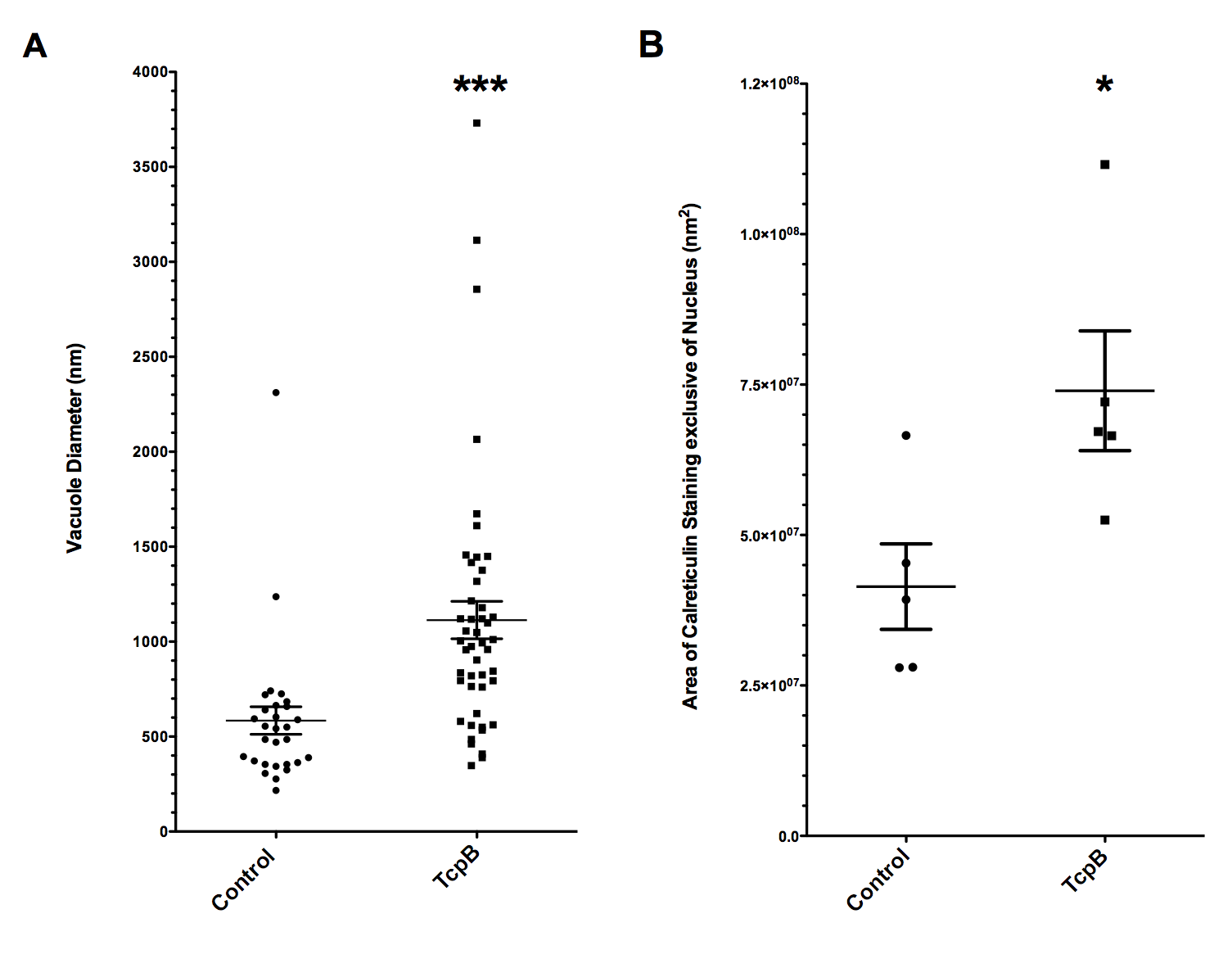

Supplement: Figure S4 — TcpB increases vacuole diameter and ER size. RAW 264.7 cells were cultured in the presence of 50 µg/ml purified TcpB for 24 hours then washed and fixed for staining as shown in Figures 7–8. Five non-dividing cells in each frame were randomly selected for quantification. Measurements were taken using the Ruler Tool within Adobe Photoshop, with the scale set at 1 pixel = 62.15 nm for 100× magnified microscopy images. A) Vacuole diameters were measured in control (29 vacuoles) and TcpB treated (47 vacuoles) cells. TcpB treatment increased vacuole diameter significantly (***P≤0.0003). B) Calreticulin staining area was assessed by measuring the entire anti-calreticulin labeled fluorescent area and then subtracting out the area of the nuclei. Calreticulin area was significantly increased in TcpB treated cells (*P≤0.03), while nuclear size was equivalent to the control cells (data not shown). (TIF) [file ppat.1003785.s004.tif]

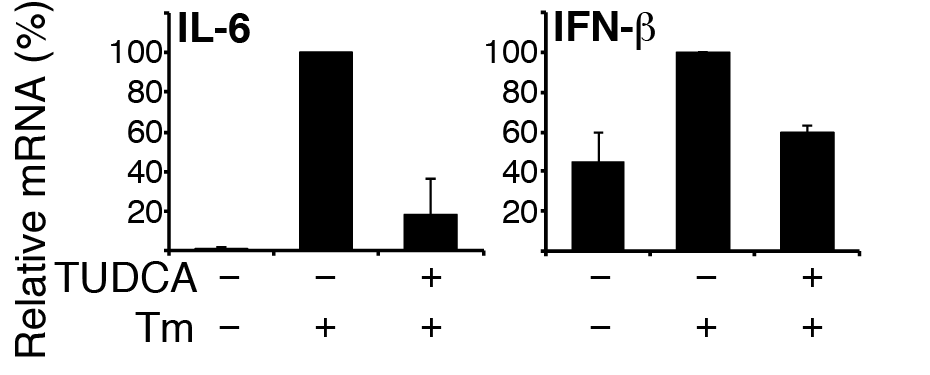

Supplement: Figure S5 — TUDCA inhibits cytokine induction by the ER stressor tunicamycin. RAW264.7 macrophages were pre-treated with 500 µg/mL TUDCA 30 min., followed by 6 h 10 µg/mL tunicamycin (Tm) as indicated, and then harvested for RNA. Relative cytokine gene expression was assessed by qPCR. Results are combined from 2 independent experiments. (TIF) [file ppat.1003785.s005.tif]

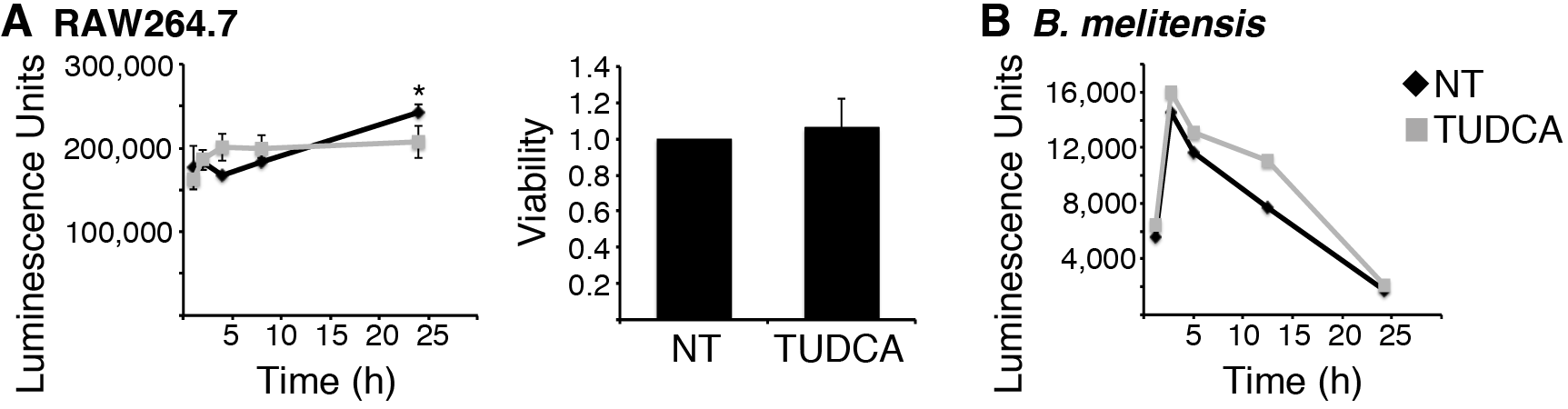

Supplement: Figure S6 — Minimal effects of TUDCA on host cell and pathogen viability. A) left panel: RAW 264.7 macrophages were treated with 500 µg/mL for the times indicated. Viability (ATP content) was determined by Cell-titer glo assay. Error bars represent standard deviation of duplicate (non-treated control, black diamonds) and triplicate (TUDCA, gray squares) determinations. Results are from an experiment showing the greatest effect of TUDCA out of 4 independent experiments. *p = 0.04. Right panel: Bars depict average of 4 experiments at the 24 h time point, normalized to untreated control (set = 1.0). B) B. melitensis in broth culture were untreated (NT) or treated with TUDCA as above for times indicated and ATP content determined by Cell-titer glo assay. (TIF) [file ppat.1003785.s006.tif]

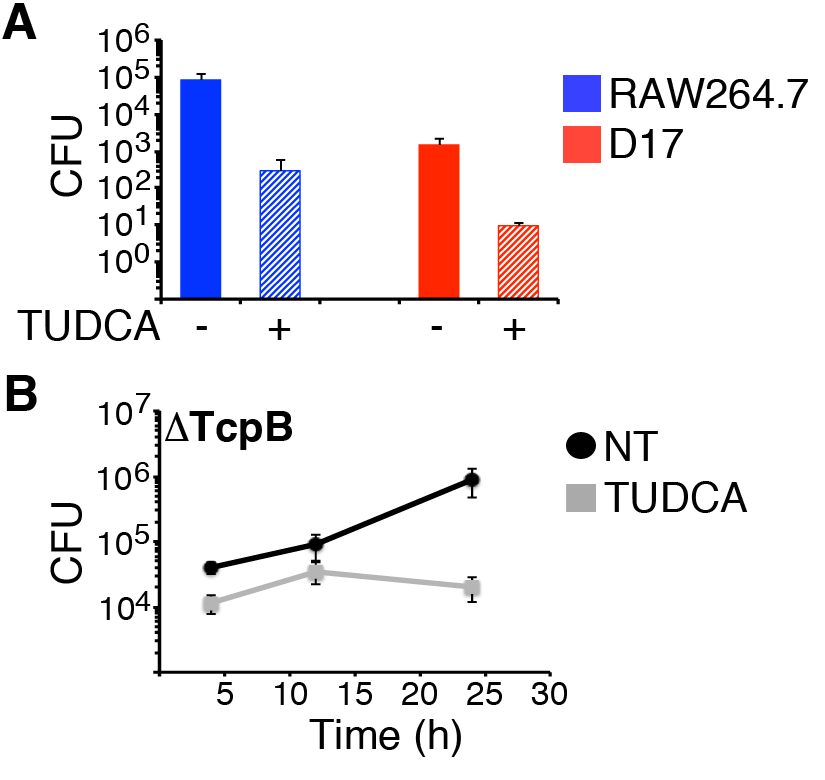

Supplement: Figure S7 — TUDCA inhibits Brucella replication in both RAW264.7 macrophages and D17 cells. A) RAW cells (blue) or D17 cells (red) were not treated (solid) or pre-treated with 500 µg/mL TUDCA (striped) for 30 minutes, infected with 100 MOI of B. melitensis, and lysed at 16 h following infection. CFU (colony forming units) were determined by transfer to dilution plates. Error bars are standard deviation of triplicate determinations. *P = 0.02. B) RAW cells were untreated (black circles) or pre-treated with 500 µg/mL TUDCA as above (gray squares), and then infected with 10 MOI of the ΔTcpB mutant Brucella. CFU were determined as in (A). Error bars represent standard deviation of 4 determinations, *p<0.04, **p<0.006. (TIF) [file ppat.1003785.s007.tif]
